# Anti-Inflammatory Evaluation of Pyrazino[2,1-*b*]quinazoline-3,6-dione Derivatives Inspired by Fiscalin B

**DOI:** 10.3390/ph19050775

**Published:** 2026-05-15

**Authors:** Márcia S. Martins, Madalena M. M. Pinto, Isabel F. Almeida, Maria T. Cruz, Emília Sousa

**Affiliations:** 1 Laboratório de Química Orgânica e Farmacêutica, Departamento de Ciências Químicas, Faculdade de Farmácia, Universidade do Porto, 4050-313 Porto, Portugal; up201605865@ff.up.pt (M.S.M.); madalena@ff.up.pt (M.M.M.P.); 2 CIIMAR—Centro Interdisciplinar de Investigação Marinha e Ambiental, 4450-208 Matosinhos, Portugal; 3 UCIBIO, i4HB, Faculdade de Farmácia, Universidade do Porto, Rua de Jorge Viterbo Ferreira, 228, 4050-313 Porto, Portugal; ifalmeida@ff.up.pt; 4 Laboratory of Pharmaceutical Technology, Faculty of Pharmacy, University of Porto, 4050-313 Porto, Portugal; 5 Faculty of Pharmacy, University of Coimbra, 3004-531 Coimbra, Portugal; trosete@ff.uc.pt; 6 CNC—Center for Neuroscience and Cell Biology, University of Coimbra, 3004-504 Coimbra, Portugal

**Keywords:** fiscalin B derivatives, neurokinin-1 receptor, anti-inflammatory activity, nitric oxide, iNOS, molecular docking, pruritus, skin inflammation

## Abstract

**Background/Objectives:** Chronic inflammatory skin diseases are frequently associated with pruritus, in which the neurokinin-1 receptor (NK_1_R) and its ligand substance P (SP) play a central role. The development of compounds combining anti-inflammatory and antipruritic effects represents a promising therapeutic strategy. This study aims to identify fiscalin B derivatives as anti-inflammatory agents with high affinity to NK_1_R using an integrated in silico and in vitro approach. **Methods:** A library of fiscalin B derivatives was screened through molecular docking against NK_1_R to identify high-affinity ligands. Selected compounds were further evaluated using in silico ADMET and toxicity predictions. In vitro assays were conducted in HaCaT keratinocytes, RAW264.7 macrophages, and NIH/3T3 fibroblasts to assess cytotoxicity, nitric oxide production, inflammatory proteins expression, and cell migration. **Results:** Docking studies identified several derivatives with predicted binding affinities comparable to or exceeding those of aprepitant, a well-established NK_1_R antagonist. Several compounds, particularly **2**, **3**, **4**, **6**, and **7**, reduced lipopolysaccharide-induced nitric oxide production to 41–51% without relevant cytotoxicity. This effect was associated with reduced iNOS protein levels, suggesting modulation of inflammatory pathways rather than direct nitric oxide scavenging. Most compounds showed positive safety profiles, although in silico analysis indicated limited biodegradability and potential aquatic toxicity. **Conclusions:** The fiscalin B derivatives, **2**, **3**, and **4**, demonstrate potential as anti-inflammatory agents, in vitro, and as NK_1_R high affinity ligands, in silico. These findings support their potential as lead compounds for topical therapies for inflammatory skin disorders associated with pruritus, although further optimization and validation are required.

## 1. Introduction

The skin is an active immunological barrier that plays a crucial role in host defense [1]. Upon exposure to inflammatory stimuli, pattern recognition receptors become activated, triggering the nuclear factor kappa B (NF-κB) and mitogen-activated protein kinase (MAPK) signaling pathways [2,3]. Activation of these pathways results in the production of pro-inflammatory mediators and the amplification of the inflammatory response. Importantly, NF-κB and MAPK signaling cascades are highly interconnected, forming positive feedback loops that can sustain and exacerbate chronic inflammation [2,3]. Pruritus is a complex and debilitating symptom frequently associated with cutaneous inflammation. Among the key mediators involved, the neuropeptide substance P (SP) and its high-affinity receptor, the neurokinin-1 receptor (NK1R), play central roles [4]. SP is released from sensory nerve endings, immune cells, and keratinocytes, and binds to NK_1_R, thereby promoting neurogenic inflammation and transmitting itch signals to the central nervous system [4]. Both inflammation and pruritus are hallmark features of numerous dermatological disorders, ranging from highly prevalent conditions such as atopic dermatitis and psoriasis vulgaris to rare autoimmune diseases, including bullous pemphigoid, pemphigus, and dermatomyositis [5]. Consequently, the development of therapeutic agents with dual anti-inflammatory and antipruritic activity represents a promising strategy for the treatment of inflammatory skin diseases associated with pruritus. However, the management of chronic pruritus presents a significant clinical challenge due to the itch–scratch cycle. Persistent scratching leads to mechanical skin damage, with excoriations and disruption of the epidermal barrier, ultimately impairing skin homeostasis [4]. Therefore, an optimal topical therapy should not only attenuate inflammation and pruritus but also maintain the integrity of the skin’s endogenous regenerative and repair mechanisms.

Corticosteroids, oral and topical, continue to be the mainstream therapy because of their potent anti-inflammatory, anti-proliferative, and immunosuppressive effects [6]. However, prolonged corticosteroid use is associated with numerous adverse effects, including inhibition of epidermal lipid synthesis, impaired antimicrobial function, skin atrophy, telangiectasia, and further barrier disruption, which restricts their use to limited durations and areas [7,8]. Other topical options include calcineurin inhibitors and phosphodiesterase-4 inhibitors, which offer fewer side effects and suitability for long-term use [9], but they are often less effective in severe lesions and can cause application-site burning or irritation [10,11]. Systemic and topical Janus kinase inhibitors have recently emerged as effective options for moderate-to-severe inflammatory dermatoses by targeting cytokine receptor signaling [12]. However, they have been associated with several adverse effects, cardiovascular conditions, cancer and serious infections [13]. In recent years, new biological therapies have emerged, yielding promising results. Specifically, biopharmaceuticals such as dupilumab, lebrikizumab, nemolizumab, and trakinumab have shown significant potential [14,15]. While these offer high specificity and efficacy, they require parenteral administration, which can be a barrier to patient compliance and carries high economic costs [15]. Aprepitant, a NK1R antagonist clinically approved for its antiemetic properties, already demonstrated capacity in reducing pruritus in several skin conditions; however inconsistent results were obtained [4].

The publication of the high-resolution crystal structures of NK_1_R in complex with the antagonist aprepitant revealed detailed features of the receptor’s hydrophobic binding pocket [16]. These insights have significantly advanced structure-based drug design approaches, enabling the rational optimization of ligand scaffolds to improve affinity and selectivity. Within this framework, fiscalin B, a marine-derived alkaloid isolated from *Neosartorya fischeri*, presents a compelling starting point. Originally identified as a SP inhibitor with an inhibitory constant value of 174 μM [17], the alkaloid fiscalin B feature a rigid pyrazino[2,1-*b*]quinazoline-3,6-dione core fused to an indole moiety. While its antitumor [18,19,20], antimicrobial [21,22], and antimalarial activities [23] were already documented, its anti-inflammatory potential remains largely unexplored, making this the first study to evaluate such activity. Structurally, fiscalin B contains quinazolinone and indole moieties, both of which have been associated with anti-inflammatory properties [24,25,26,27]. By merging these intrinsic anti-inflammatory motifs with the molecule’s intrinsic NK_1_R inhibitory activity, this scaffold offers a good starting point for the development of potential dual-activity compounds, anti-inflammatory and antagonistic activity of NK_1_R. Among the multiple pathways involved in inflammation and pruritus, the SP/NK_1_R axis is particularly attractive due to its dual role in both neurogenic inflammation and itch transmission [28]. This makes NK_1_R a relevant target to address both processes simultaneously. Additionally, the availability of high-resolution NK_1_R crystallographic structures supports structure-based drug design, further justifying its selection over other targets.

The primary objective of this study is to identify hit compounds from an in-house library of fiscalin B derivatives with dual pharmacological activity, namely high-affinity NK_1_R antagonism and potent anti-inflammatory effects. To achieve this goal, an integrated screening approach was employed, combining molecular docking and virtual screening with subsequent in vitro evaluation of anti-inflammatory activity. Cytotoxicity assays in skin-representative cells and an assessment of the compounds’ effect on cell migration were also performed.

## 2. Results and Discussion

### 2.1. Virtual Screening of a Library of Fiscalin B Derivatives

A virtual screening was carried out with a library of fiscalin B derivatives. Numerous compounds with the potential to bind to NK_1_R were identified. From this library, 5 molecules (**1**, **2**, **3**, **4**, and **5**) were found to have a lower or equal free energy of binding to NK_1_R than aprepitant (−10.9 kcal·mol^−1^), a NK_1_R antagonist clinically approved for its anti-emetic properties. These, along with 12 additional molecules, exhibited binding energies superior or equivalent to fiscalin B (−10.1 kcal·mol^−1^), a marine natural product described as a NK_1_R antagonist [17] (Table 1). In the case of Autodock Vina, the score corresponds to the estimated binding free energy. The free energy difference between the diastereomers **2** and **4** is less than 0.2 (kcal·mol^−1^). These results support that, within the reported accuracy of docking calculations with AutoDock Vina (3 kcal/mol) [29], there should be no significant diastereoselectivity. The remaining compounds were found to have docking scores between −9.4 and −10.8 kcal·mol^−1^. The best five compounds, together with fiscalin B, were selected for functional studies.

**Figure 1:**
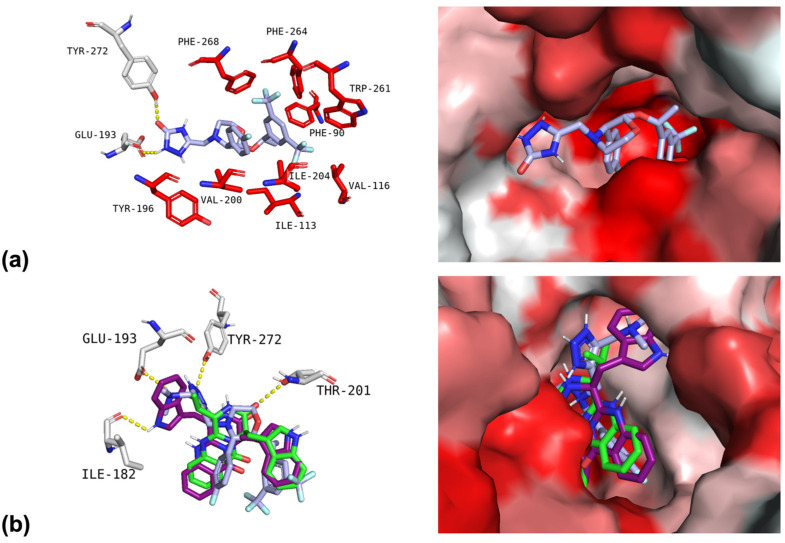
(**a**) Interactions of aprepitant with the neurokinin 1 receptor (NK_1_R) binding site. The left panel represents the polar (hydrogen bonds) and hydrophobic interactions of aprepitant with the binding site amino acids. The right panel displays aprepitant docked within the NK_1_R binding site (surface representation). (**b**) Interactions of aprepitant (light purple), fiscalin B (green), and compound **1** (dark purple) with the NK_1_R binding site. The left panel is a visual representation of the hydrogen bond interactions of the three compounds with the active site. The right panel displays the three compounds docked within the NK_1_R binding site (surface representation). The NK_1_R active site amino acid residues involved in polar interactions are shown in grey sticks. Amino acid residues involved in hydrophobic interactions are shown in red sticks. Polar interactions (hydrogen bonds) are indicated by yellow dashed lines. Nitrogen and oxygen atoms in the ligands are represented as blue and red, respectively. The NK_1_R binding site is shown in a surface gradient of red (redder indicates higher hydrophobicity). Residues involved in the interactions are labelled: TYR-tyrosine; PHE-phenylalanine; TRP-tryptophan; VAL-valine; ILE-isoleucine; GLU-glutamic acid; THR-threonine.

A visual inspection of the binding conformations for NK_1_R was performed for the top five compounds, together with fiscalin B and aprepitant, to interpret the calculated binding free energies using the PyMol software. The NK_1_R binding site is characterized by a narrow, hydrophobic pocket primarily composed of residues from the transmembrane helices. The key residues forming this cleft include Tyr272, Ile204, Ile113, Phe264, Phe268, Trp184, Ile182, Trp261, Val200, Tyr196, Gln165, Asn109, Thr201, Pro112, Glu193, His265, His197, and His108. Figure 1a illustrates the best binding pose for the aprepitant-NK_1_R complex. Aprepitant fits deeply into the hydrophobic pocket, establishing polar interactions (hydrogen bonds) with Tyr272 and Glu193. Additionally, it can form several significant hydrophobic interactions (within 4 Å) with surrounding non-polar residues, including Tyr196, Val200, Phe268, Phe264, Phe90, Trp261, Val116, Ile113, and Ile204.

Fiscalin B and its highest-scoring derivative, **1**, occupy the same region within the hydrophobic cleft as aprepitant. The superimposed image in Figure 1b reveals strong overlap between the core structures. The quinazolinone-fused diketopiperazine core of the compounds aligns with the 2,3,4-substituted-morpholine portion of aprepitant. The 4-fluorophenyl group of the fumiquinazoline derivatives occupies the region corresponding to aprepitant’s 3,5-bis(trifluoromethyl)benzene group. The other substituent of fiscalin B (isopropyl) and compound **1** (indole) are oriented towards aprepitant’s triazolinone portion. The polar interactions established by the compounds showed several differences. Aprepitant establishes two hydrogen bonds in the triazolinone region, one between its carbonyl group and Tyr272, and a second between an amine group and Glu193. Fiscalin B establishes only one hydrogen bond between the carbonyl group of its amide moiety and Thr201. The structural modification from fiscalin B (isopropyl group) to compound **1** (indole group) prevents the hydrogen bond with Thr201 but instead facilitates the formation of a new hydrogen bond with Ile182. This shift in polar contacts, from the hydroxyl group of Thr201 to the backbone amine of Ile182, may partially explain the improved binding affinity observed for compound **1**, suggesting a favourable repositioning of the ligand’s core within the binding cleft and an overall optimization of the interaction pattern compared to the parent fiscalin B.

**Figure 2:**
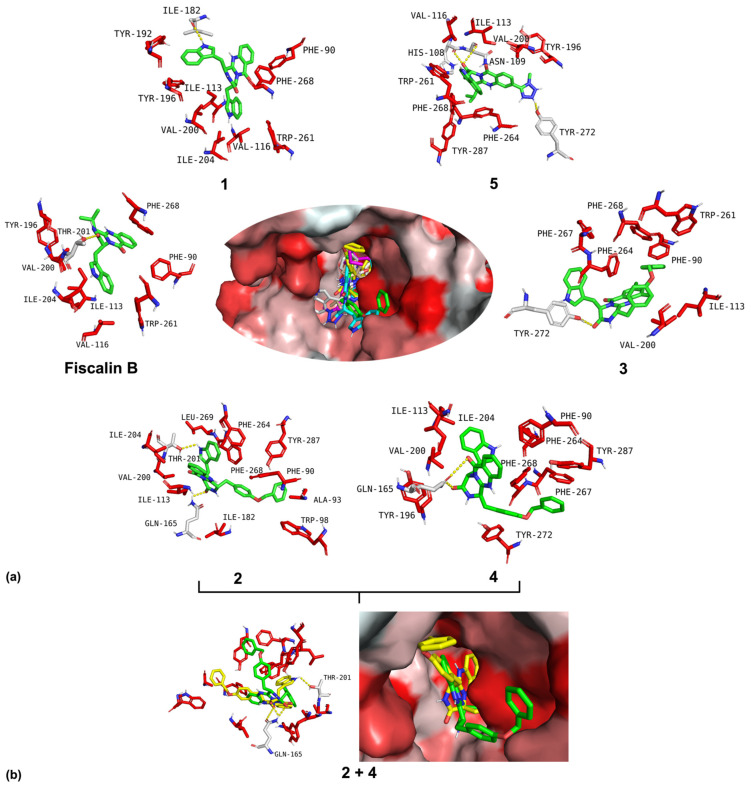
(**a**) Interactions of fiscalin B (center-left panel) and its derivatives **1**, **2**, **3**, **4**, and **5** (surrounding panels) with the neurokinin 1 receptor (NK_1_R) binding site. The central figure displays the superimposed binding poses of the six fiscalins docked into the NK_1_R binding pocket (surface representation). All compounds are represented in green sticks in the individual interaction panels. The NK_1_R active site amino acid residues involved in interactions are shown in red sticks (apolar) and grey sticks (polar). Yellow dashed lines indicate polar interactions (hydrogen bonds). Nitrogen and oxygen atoms in the ligands are represented as blue and red, respectively. The NK_1_R binding site is shown in a surface gradient of red (redder indicates higher hydrophobicity). (**b**) Visual representation of the binding form of the two diastereomers, **2** (represented in green sticks) and **4** (represented in yellow sticks), in the NK_1_R binding site. The left image shows the key interactions of **2** and **4** with the surrounding residues (red and grey sticks), while the right image shows their superimposed binding poses in the hydrophobic pocket. Residues involved in the interactions are labelled: TYR-tyrosine; PHE-phenylalanine; TRP-tryptophan; VAL-valine; ILE-isoleucine; GLU-glutamic acid; THR-threonine; GLN-glutamine; ALA-alanine; LEU-leucine.

The interactions of the best five fiscalin B derivatives were further analyzed (Figure 2a). All compounds fit into the hydrophobic pocket of NK_1_R and establish both hydrogen bond and non-polar interactions with the receptor, as detailed in Table S1 (Supporting Information). Compound **4** establishes a hydrogen bond between the amine of the tryptophan moiety and Thr201, and between the carbonyl of the amide group and Gln165. The carbonyl of the amide of compound **3** establishes a hydrogen bond with Tyr272. Derivative **4** establishes a double interaction with the same amino acid residue, Gln165, using both carbonyls of the quinazolinone core. Compound **5** establishes three polar interactions, one with the 1-methyl-1*H*-tetrazol-5-yl substituent and Tyr272, and the others with the amide group and His108 and Asn109. Beyond traditional hydrophobic contacts, the numerous aromatic residues lining the NK_1_R pocket suggest that π-π stacking interactions are potentially a major stabilizing force. The analyses of the poses (Figure 1a and Figure 2a) confirm that the quinazolinone core and the 4-fluorophenyl group engage in significant edge-to-face (T-shaped) or parallel-displaced π-interactions with amino acid residues such as Phe268, Phe264, and Trp261. The non-polar interaction profile detailed in Table S1 reveals that the hydrophobic residues Phe268, Ile113, and Val200 are potentially interacting with all seven tested compounds. This involvement, coupled with the wide range of other hydrophobic interactions, supports the hydrophobic nature of the receptor and suggests that these three residues might be important for hydrophobic interactions necessary for binding to the NK_1_R pocket.

The difference in chirality between diastereomers **2** and **4**, despite their similar docking scores, results in different polar interactions. As noted in Table S1, derivative **2** maintains the Thr201 interaction characteristic of fiscalin B but adds a hydrogen bond with Gln165. In contrast, **4** loses the Thr201 interaction and instead establishes a new double H-bond interaction with Gln165. Visually (Figure 2b), this difference in chirality is accomplished by orienting the aromatic substituent *O*-benzyl-L-tyrosine differently in the receptor pocket, leading to distinct hydrophobic contacts. A comparison of **2** and **3** reveals the impact of adding two chlorine substituents to the quinazolinone core. Although the docking scores are similar, the introduction of halogens significantly alters the compound’s binding motif. The tricyclic core of the fumiquinazoline derivatives is relatively rigid, meaning the addition of the chlorine atoms in derivative **3** does not significantly change the compound’s intrinsic conformation. However, since the NK_1_R pocket is narrow, the increased steric hindrance introduced by the two halogens forces the compound to reorient within the binding cleft. This subtle reorientation causes the molecule to lose the Thr201 and Gln165 polar contacts and instead forms a H-bond with Tyr272, thereby mimicking a key polar contact made by the clinical antagonist aprepitant. Furthermore, the chlorine atoms themselves are highly lipophilic, acting as favourable hydrophobic interaction points within the binding pocket. This enhanced lipophilicity and capacity for halogen bonding can explain why the overall docking score remains consistent between the halogenated **3** and the non-halogenated **2**.

Our computational results provide a structural rationale for the previously reported biological activity of fiscalin B as an NK_1_R antagonist [17]. By successfully docking into the same hydrophobic cleft as the clinical antagonist aprepitant, fiscalin B demonstrates a plausible competitive binding mechanism against the receptor’s endogenous agonist. This structural insight not only corroborates its known pharmacological profile but also establishes a baseline for molecular optimization. Consequently, given that five compounds (**1**, **2**, **3**, **4**, and **5**) demonstrated estimated binding free energies equal to or better than the clinical control (aprepitant, −10.9 kcal·mol^−1^), these findings highlight their potential as novel NK_1_R antagonists. The next critical step involves experimental validation of these computational predictions using, for example, competitive binding assays using a known binding ligand [^3^H] SP or cell-based Ca^2+^ mobilization assays against NK_1_R.

While molecular docking provides a valuable structural hypothesis for the binding of these derivatives to the NK_1_R, the methodology has inherent limitations. Standard protocols employ rigid receptor models, treating the highly dynamic NK_1_R as a rigid structure and failing to capture essential induced-fit conformational changes upon ligand binding [16]. Thermodynamically, scoring functions often prioritize enthalpy over entropic penalties and overlook the complex role of desolvation and structural water molecules. Additionally, these simulations generally ignore the influence of the surrounding lipid bilayer on receptor conformation and cannot estimate ligand residence time, a key factor in pharmacological efficacy. Consequently, these results serve as a preliminary screening tool that requires experimental validation to confirm functional potency and the specific nature of the pharmacological response.

### 2.2. Prediction of Pharmacokinetic Properties, Aquatic Toxicity, Biodegradability, and Targets Related to Inflammation

The pharmacokinetic properties of the 18 derivatives with higher or equal binding affinity than fiscalin B, together with the reference compound aprepitant, were evaluated using in silico methodologies to estimate parameters particularly relevant for topical delivery, namely molecular weight, octanol–water partition coefficient (log P), skin permeation coefficient (log Kp), and the probability of skin sensitization and eye irritation (Table S2, Supporting Information). Skin permeation through epidermis is largely affected by molecular weight and lipophilicity, with commonly accepted criteria for passive diffusion being a molecular weight below 500 Da and a log P between 1 and 4. Most fiscalin B derivatives met the molecular weight criteria, except compounds **2**, **3**, **4**, **17**, together with aprepitant, which are therefore expected to permeate less efficiently. Log P values predominantly fell within the optimal range, whereas **2**, **4**, **8**, **15**, and **17** presented higher lipophilicity (values between 4 and 5), and compound **3** exceeded log P 5, suggesting a greater tendency for *stratum corneum* retention and a potential slower diffusion into deeper skin layers. Predicted skin sensitization was high for compounds **11** and **12**, moderate for fiscalin B, **8**, **9**, **10**, **13**, **15**, **16**, and **17**, and low for the remaining, while only compound **15** and fiscalin B being associated with a moderate probability of eye irritation, with minimal risk estimated for the others. These data indicate that, although several derivatives fulfil the basic requirements for topical delivery, some combine high lipophilicity with an unfavorable sensitization profile that would require optimization.

Given the continuous discharge of topical medicines into wastewater and the increasing concern about persistent micropollutants, the biodegradability and aquatic toxicity of the derivatives were also assessed using the EPI Suite^TM^ software (developed by the U.S. Environmental Protection Agency U.S. EPA [30]), and its BIOWIN and ECOSAR models. All derivatives exhibited slow biodegradation or were classified as recalcitrant (Table S3, Supporting Information). The least biodegradable compounds (**2**, **3**, **4**, **8**, **11**, and aprepitant) were predominantly halogenated analogues, in line with the high stability of carbon-halogen bonds. Moreover, compounds **2** and **4**, although non-halogenated, contain a benzyl protected tyrosine substituent, a protecting group intentionally designed to resist chemical transformation, which likely accounts for their lower biodegradability relative to the deprotected derivatives **6** and **7**. ECOSAR predictions indicated that all compounds fall within the slightly, moderately or very high toxic categories under acute exposure, and as moderately toxic or very highly toxic under chronic exposure. On average, fish was the most sensitive species in acute scenarios, whereas daphnids were more susceptible under chronic exposure, followed by fish and green algae. The highest predicted aquatic toxicity was observed for **2, 3**, **4**, and **8**, which are also among the most lipophilic derivatives, in agreement with ECOSAR’s reliance on log P [31]. Aprepitant exhibited comparatively high predicted toxicity. Conversely, compounds such as **5**, **12**, **13**, **14**, as well as the parent fiscalin B, exhibited comparatively lower predicted toxicity, although still within categories that warrant caution. Table S4 (Supporting Information) summarizes all the outcomes obtained using the ECOSAR model. The complete data obtained is present in Table S3 (Supporting Information).

Taken together, the in silico analysis highlights a heterogeneous profile among the tested compounds, with some candidates meeting key requirements for topical delivery but displaying limitations related to environmental safety. In particular, the combination of high lipophilicity, poor biodegradability, and predicted aquatic toxicity raises concerns regarding both dermal delivery and ecological impact. The overall low biodegradability and moderate-to-high ecotoxicity predicted for most compounds raise concerns regarding their potential persistence and impact following environmental release. These findings should therefore be considered as an early-stage screening that highlights both promising candidates and structural liabilities. Future work should therefore focus on experimental validation of skin permeation, sensitization and environmental fate, alongside further structural optimization—such as reduction in lipophilicity and the avoidance of recalcitrant moieties (e.g., halogenated substituents or persistent protecting groups)—to achieve a more balanced profile between dermatopharmacokinetic and ecotoxicological sustainability.

**Figure 3:**
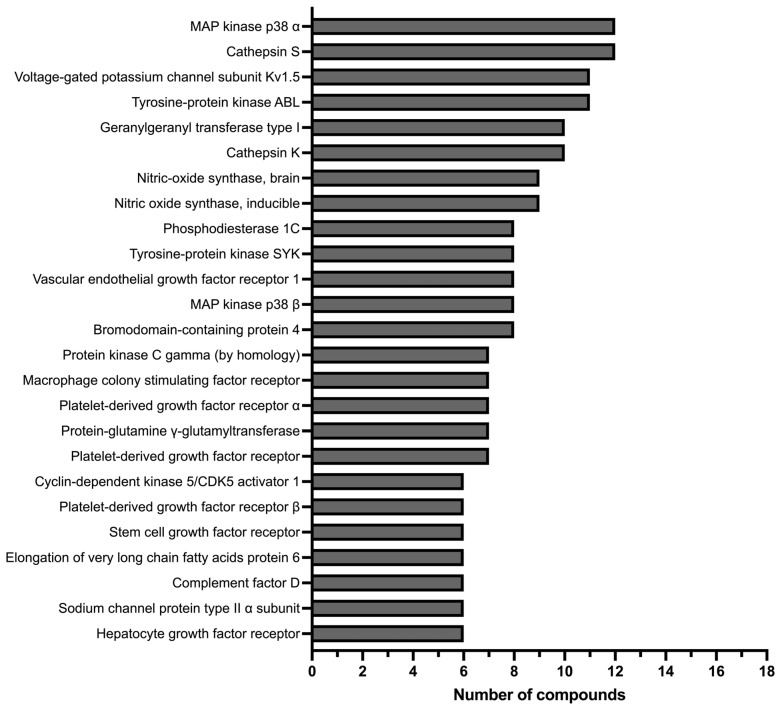
Top 25 of the predicted targets for fiscalin B derivatives using the SwissTargetPrediction web tool [32].

To obtain an idea of the biological space of these derivatives, the potential targets were predicted using the SwissTargetPrediction [32]. The selection was based on prediction frequency, with the 25 most frequently predicted targets identified and listed in Figure 3.

In silico target prediction identified multiple proteins involved in inflammatory pathways as potential targets of fiscalin B derivatives, supporting their anti-inflammatory potential. Prominent targets include MAPK α and β, key regulators of intracellular signaling leading to the production of pro-inflammatory cytokines such as TNF-α, IL-6, and IL-1β [33]. Additional targets, including cathepsins S and K, further reinforce this profile due to their roles in antigen presentation and extracellular matrix remodeling in chronic inflammation [34]. The prediction of inducible nitric oxide synthase (iNOS) also highlights a mechanism involving modulation of nitric oxide production, a critical mediator of inflammatory responses [35]. Furthermore, several proteins associated with chronic inflammation and tissue remodeling were identified, including SYK, cyclin-dependent kinase 5, and multiple growth factor receptors, which contribute to macrophage activation, fibroblast proliferation, and pathological angiogenesis [36,37,38,39]. The identification of bromodomain-containing protein 4 (BRD4) suggests an additional layer of transcriptional regulation of pro-inflammatory genes [40], while complement factor D points to potential modulation of the complement cascade [41].

Overall, these findings suggest that the fiscalin B derivatives are potential multi-target molecules capable of modulating complementary inflammatory pathways, including cytokine production, nitric oxide signalling, kinase activation, and immune cell recruitment, supporting their potential in the treatment of inflammatory diseases.

Taken together, the in silico analysis suggested that the tested compounds display mixed suitability for topical use and generally unfavorable environmental profiles, characterized by poor biodegradability and aquatic toxicity. These findings should therefore be considered as an early-stage screening that highlights both promising candidates and structural liabilities, underscoring the need for subsequent experimental assessment of dermal absorption, sensitization and environmental fate, as well as for further structural optimization aimed at improving both pharmacokinetic and ecotoxicological properties. However, predictions from the in silico platforms used in this study should be interpreted with caution. These models are primarily based on two-dimensional structural descriptors and SMILES-derived inputs and therefore may not fully capture the influence of stereochemistry, conformational flexibility, ionization state, and other three-dimensional features that can affect absorption, distribution, metabolism, and toxicity. In particular, for structurally complex scaffolds such as fiscalin B derivatives, subtle spatial differences and multiple stereocenters may contribute significantly to biological behavior while remaining indistinguishable to these predictive algorithms. Likewise, target-prediction tools offer useful hypotheses regarding possible protein interactions, but they are limited by the availability of known ligands in training databases and cannot distinguish between agonistic and antagonistic effects or provide quantitative affinity or potency values. Therefore, the computational results presented here should be considered as supportive and hypothesis-generating, and further experimental validation will be necessary to confirm the pharmacokinetic and safety profiles of these compounds.

### 2.3. Assessment of Cellular Metabolic Activity in the Human Keratinocyte HaCaT Cell Line

**Figure 4:**
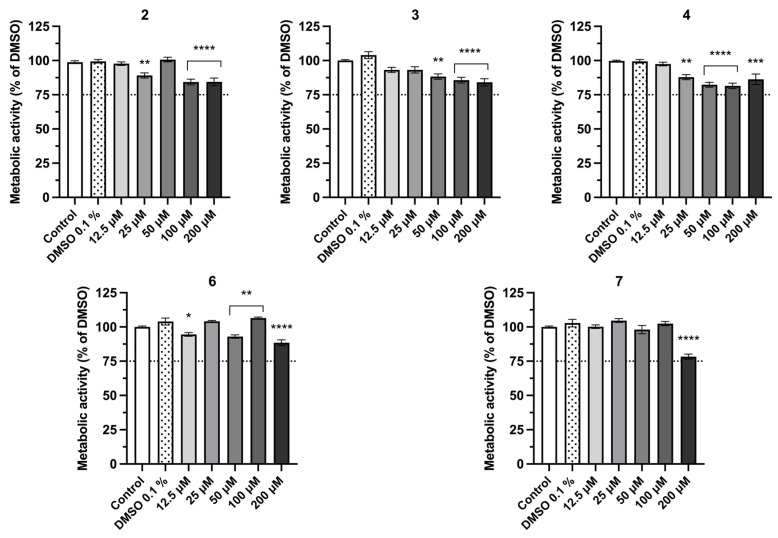
Cell metabolic activity of HaCaT cells exposed to increasing concentrations of fiscalin B derivatives **2**, **3**, **4**, **6**, and **7** (0–200 μM) for 24 h, measured by the Alamar Blue assay. Values are the mean ± SEM of at least four independent experiments performed in duplicate. Statistical comparisons were made using Ordinary One-way ANOVA followed by Dunnett’s multiple comparisons test (* *p* < 0.05, ** *p* < 0.01, *** *p* < 0.001, **** *p* < 0.0001 vs. DMSO 0.1%).

Considering that topical formulations targeting inflammation need to permeate the epidermis to reach the target receptors, the cytotoxicity of the compounds was evaluated using a human keratinocyte (HaCaT) cell line (Figure 4). For the comprehensive screening data across the entire library, please refer to Figure S1 in the Supporting Information. The used vehicle, dimethyl sulfoxide (DMSO) at a concentration of 0.1%, did not significantly affect cellular metabolism relative to the untreated cells (control). Synthetic fiscalin B and derivatives were tested at concentrations ranging from 12.5 μM to 200 μM. According to the international standard ISO 10993-5, a test material is considered cytotoxic if it causes a reduction in cell metabolic activity by more than 30% relative to control cells cultivated in cell culture medium, meaning that a viability threshold of 70% or higher corresponds to a non-cytotoxic effect [42]. Therefore, a more conservative viability threshold of 75% was established to determine compound eligibility for subsequent in vitro assays.

Among the 18 tested derivates, five compounds demonstrated cytotoxicity: compound **1** at 200 μM, compound **10** at concentrations superior to 25 μM, compound **14** at 200 μM, compound **15** at concentrations superior to 50 μM, and compound **16** at 100 and 200 μM. Based on these results, compounds **1** (200 µM), **10** (>25 µM), **14** (200 µM), **15** (>50 µM), and **16** (>50 µM) were excluded from further evaluations due to cytotoxicity concerns.

### 2.4. Assessment of Cytotoxicity and Anti-Inflammatory Activity in RAW 264.7 Macrophage Cells

Given that compounds must penetrate the epidermis to reach the dermis and exert their anti-inflammatory effects, it is crucial to assess their cytotoxicity in immune cells. Macrophages are key innate immune cells involved in regulating and modulating inflammatory responses [43]. Thus, the effect of the derivatives on cellular metabolism was evaluated in the murine macrophage cell line RAW 264.7 to determine non-toxic concentration ranges. These concentrations were subsequently applied in anti-inflammatory assays, targeting the reduction in NO following stimulation with lipopolysaccharide (LPS), a Toll-like receptor 4 agonist. The library of synthetic pyrazino[2,1-*b*]quinazoline-3,6-diones, including the fiscalin B synthetic analogue, was selected for initial studies.

**Figure 5:**
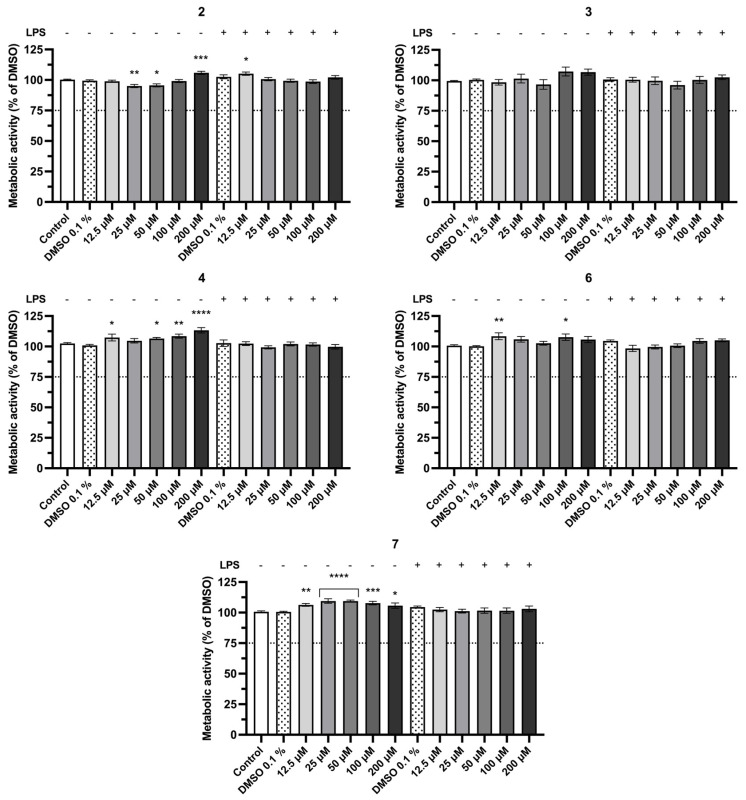
Cell metabolic activity of RAW 264.7 cells exposed to increasing concentrations of fiscalin B derivatives **2**, **3**, **4**, **6**, and **7** (0–200 μM) alone or followed by LPS (100 ng·mL^−1^), for 24 h, measured by the Alamar Blue assay. Values are the mean ± SEM of at least four independent experiments performed in duplicate. Statistical comparisons were made using Ordinary One-way ANOVA followed by Dunnett’s multiple comparisons test (* *p* < 0.05, ** *p* < 0.01, *** *p* < 0.001, **** *p* < 0.0001 vs. DMSO 0.1%).

**Figure 6:**
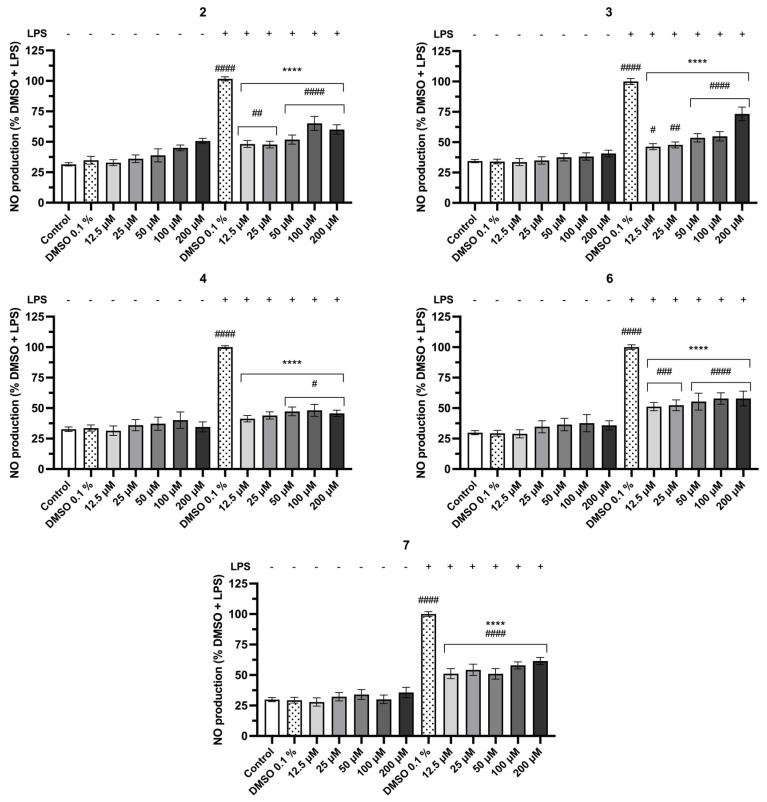
Effect of fiscalin B derivatives **2**, **3**, **4, 6**, and **7** on nitric oxide (NO) production. Nitrite levels were determined using the Griess assay in RAW 264.7 cells exposed to fiscalin B derivatives (12.5–200 μM) in the absence or in the presence of LPS for 24 h. Values are the mean ± SEM of at least three independent experiments performed in duplicate. Statistical comparisons were made using Ordinary One-way ANOVA followed by Dunnett’s multiple comparisons test (**** *p* < 0.0001 vs. DMSO 0.1% + LPS; ^#^ *p* < 0.05, ^##^ *p* < 0.01, ^###^ *p* < 0.001, ^####^ *p* < 0.0001 vs. DMSO 0.1%).

RAW 264.7 cells were exposed to synthetic fiscalin B and its derivatives in concentrations ranging from 12.5 µM to 200 µM for 24 h, with or without LPS stimulation applied 60 min after compound addition. Due to cytotoxicity observed in the keratinocyte cell line, compounds **1** and **14** were not tested at 200 µM, nor were **10** (>25 µM), or **15** and **16** (>50 µM). Cellular metabolic activity (Figure 5) and nitrite production (Figure 6) were determined using the Alamar Blue and the Griess assay, respectively. The full screening dataset for all library members is provided in Figures S2 and S3 (Supporting Information). Analyzing the obtained data regarding metabolic activity (Figure 5), it can be observed that the used vehicle, DMSO at the concentration of 0.1%, did not significantly affect the cell metabolic activity when compared to untreated cells (control). Similar to the HaCaT cell line assay, a threshold of 75% was applied to disclose cytotoxicity. Based on this criterion, only four compounds—**10**, **15**, and **16** (at concentrations above 25 µM), and **17** (at concentrations above 50 µM)—induced a reduction in cellular metabolic activity greater than 25%, excluding these concentrations from further testing. For all other compounds, the observed decrease in metabolic activity was below 25%, allowing their inclusion in subsequent experiments. Previous studies in a differentiated SH-SY5Y neuronal cell line revealed that compounds **6**, **7**, **8**, and **16** were cytotoxic at concentrations ≥ 25 µM, whereas compounds **3**, **15**, and **17** were cytotoxic across all tested concentrations (5–50 µM). Nevertheless, for concentrations ≤ 10 µM, several compounds maintained cell viability above 75% [44]. Based on these results, it can be inferred that mono-halogenation of the quinazolinone moiety increases cytotoxicity, while di-halogenation of this structural portion does not produce significant alterations in cellular metabolism. This suggests that halogenation pattern significantly modulates fiscalin B derivatives’ safety profile.

The addition of LPS did not significantly affect the cellular metabolic activity (Figure 5) but induced a robust increase in NO production (Figure 6), as expected. Regarding NO production, none of the compounds affected NO levels per se. In contrast, the majority of the compounds significantly reduced LPS-induced NO production. Notably, compounds **2**, **3**, **4**, **6**, and **7** reduced NO levels for 41.3%, 51.1%, 48.2%, 51.2%, and 46.0%, respectively, at the lowest tested concentration (12.5 µM), with this inhibitory effect remaining consistent across the full concentration range tested (12.5–200 µM), suggesting a concentration-independent mechanism. Compounds **10**, **16**, **15**, and **17** also produced a significant and concentration-dependent reduction in NO production to 65.6%, 66.7%, 56.5%, and 54.2%, respectively. In contrast, compound **14** and fiscalin B elicited a significant increase in NO production after the LPS stimulus at the lowest concentrations tested (12.5 and 25 µM), an effect not observed in the absence of LPS. This finding suggests a possible interaction between these compounds and LPS and/or the respective receptor TLR4, specifically at low concentrations. At elevated concentrations (≥50 µM for fiscalin B and ≥100 µM for **14**), both compounds caused a significant decrease in NO production. The remaining compounds, fiscalin B, **1**, **8**, **9**, **11**, **12**, and **13** also demonstrated the ability to reduce the production of NO when compared to DMSO 0.1% + LPS; however, higher concentrations were required. Compound **5** was the only one that did not significantly affect NO levels, indicating an absence of anti-inflammatory activity in this assay.

To the best of our knowledge, reports documenting the anti-inflammatory activity of pyrazino[2,1-*b*]quinazoline-3,6-dione derivatives remain scarce. However, very recently the isolation of (±)-17-hydroxybrevianamide N from the fungus *Aspergillus* sp. (CHNSCLM-0151) led to the disclosure of its strong inhibitory activity against NO production in LPS-induced RAW264.7 cells. In the same study, a series of semi-synthetic derivatives was generated by modifying the imide, phenolic hydroxyl, and carbonyl groups. The most potent derivative, bearing a propylene substituent at the C-17 position, inhibited NO production with a half-maximal inhibitory concentration (IC_50_) of 0.5 μM. Furthermore, this lead compound significantly suppressed the secretion of TNF-α, IL-6, and IL-1β. Mechanistic studies revealed that its activity is mediated by the downregulation of MAPK signaling (phosphorylation of p38, ERK, and JNK) and potent inhibition of the NF-κB pathway, specifically by suppressing IκB-α phosphorylation and blocking the nuclear translocation of p65 [45]. Other alkaloids sharing structural similarities with the pyrazino[2,1-*b*]quinazoline-3,6-dione core have also demonstrated significant anti-inflammatory potential. Oxepinamide A, a natural product featuring a fused pyrimidinone core, exhibited good topical anti-inflammatory activity in the resiniferatoxin-induced mouse ear edema assay (a model of neurogenic inflammation), inhibiting edema by 82% at a dose of 50 μg/ear [46]. Circumdatin-type alkaloids (quinazoline benzodiazepines) isolated from coral-associated fungi showed potent inhibition of LPS-induced NO production and NF-κB reporter gene activation, with circumdatin D emerging as the most promising alkaloid [47]. Moreover, isaindigotone derivatives (pyrroloquinazolines) were shown to reduce prostaglandin E_2_ and NO generation in LPS-stimulated macrophages [48]. Subsequent studies clarified that these effects are likely due to the dual inhibition of COX-2 and 5-lipoxygenase enzymes [49].

### 2.5. Nitric Oxide Scavenging Activity

SNAP (*S*-nitroso-*N*-acetyl-D,L-penicillamine), a NO donor, at neutral pH, undergoes spontaneous denitrosation in aqueous solution and, consequently, releases the NO radical [50]. The SNAP assay is a well-established in chemico method for evaluating the NO-scavenging capacity of compounds, offering insights into their potential antioxidant and anti-inflammatory properties [51,52].

**Figure 7:**
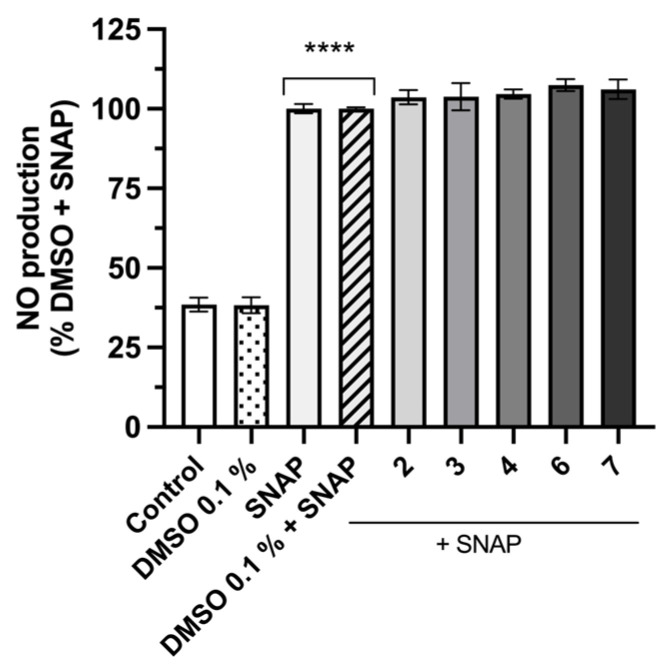
Nitric oxide (NO) scavenging potential of fiscalin B derivatives. Different compounds dissolved in dimethylsulfoxide (DMSO) 0.1% were incubated with *S*-nitroso-*N*-acetyl-DL-penicillamine (SNAP) (300 μM) in culture medium for 3 h. Results are expressed as a percentage of NO release induced by DMSO 0.1% + SNAP, the positive control. Each value represents the mean ± SEM of three experiments performed in duplicate. Statistical comparisons were made using Ordinary One-way ANOVA followed by Dunnett’s multiple comparisons test (**** *p* < 0.0001 vs. DMSO 0.1%).

In this study, the NO-scavenging activity of the most promising compounds, **2**, **3**, **4**, **6**, and **7** was assessed using the Griess reagent after incubation with SNAP at 300 µM. The results presented in Figure 7 showed no significant difference in nitrite levels compared to SNAP + DMSO 0.1%, indicating that the tested compounds do not exhibit NO scavenging activity under these conditions. This suggested that the observed reduction in NO production in the cell-based assay is unlikely to be the result of a direct chemical neutralization of NO. A lack of direct NO scavenging activity points toward alternative mechanisms of NO modulation by fiscalin B derivatives, such as downregulation of inducible nitric oxide synthase (iNOS) activity [52,53], inhibition of upstream pro-inflammatory cascade such as NF-κB suppression [54], and/or induction of antioxidant pathways (e.g., nuclear factor-erythroid 2-related factor 2 activation [55]), leading to reduced iNOS transcription, iNOS protein levels, and ultimately lower NO production [53]. The DMSO concentration in the assay system was maintained at or below 0.1%, given that higher concentrations have their own free radical scavenging and neuroprotective effects, which can confound data interpretation [56].

### 2.6. Evaluation of iNOS, pro-IL-1, and p38 MAPK Protein Levels

Fiscalin B derivatives demonstrated the capacity to reduce nitrite levels in RAW264.7 macrophages subjected to LPS stimulation. To gain deeper insight into their anti-inflammatory activity, the protein expression of two key inflammatory markers, iNOS and pro-interleukin-1 beta (pro-IL-1β), was evaluated by Western blotting. The most promising derivatives **2**, **3**, **4**, **6**, and **7** were tested at a concentration of 12.5 μM, chosen based on their cytotoxicity profiles and efficacy in inhibiting NO production.

**Figure 8:**
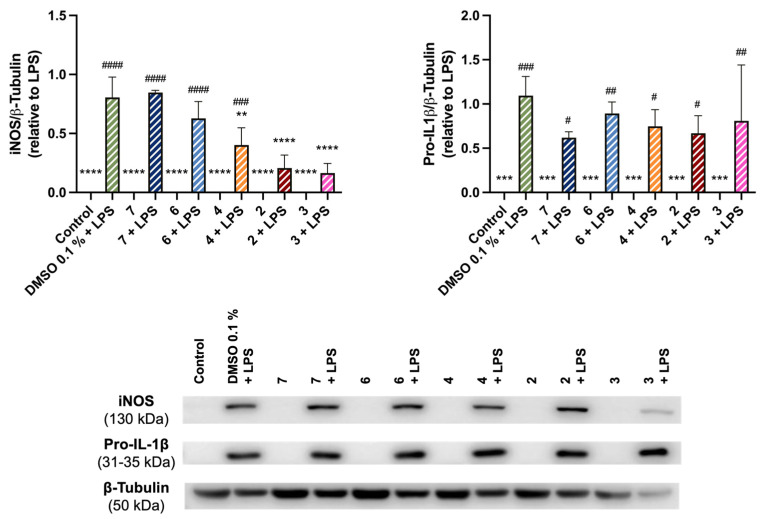
Anti-inflammatory effect of fiscalin B derivatives **2**, **3**, **4**, **6**, and **7** at the concentration of 12.5 μM in LPS-activated RAW 264.7 cells. The protein levels of iNOS and pro-IL-1β were determined in RAW 264.7 cells activated by LPS and exposed to the derivatives for 24 h, by Western Blotting. Representative images of the blots are shown, and the same loading control, β-tubulin, was used for both measured proteins. Data correspond to the mean ± SEM of three independent experiments. Statistical comparisons were made using Ordinary One-way ANOVA followed by Dunnett’s multiple comparisons test (** *p* < 0.01, *** *p* < 0.001, **** *p* < 0.0001 vs. DMSO 0.1% + LPS; (^#^ *p* < 0.05, ^##^ *p* < 0.01, ^###^ *p* < 0.001, ^####^ *p* < 0.0001 vs. DMSO 0.1%). Legend: Control—untreated cells; LPS + DMSO 0.1%—LPS-activated cells treated with 0.1% DMSO; “Compound”—cells treated with the fiscalin B derivative; “Compound” +LPS—LPS-activated cells treated with the fiscalin B derivative.

Analysis of Figure 8 shows that treatment with DMSO + LPS increased iNOS protein levels, as expected given iNOS’s role in NO synthesis. Co-treatment with compounds **2**, **3**, and **4** resulted in a significant decrease in iNOS levels compared to DMSO + LPS, with the effect more pronounced for **2** and **3**. In contrast, compounds **6** and **7** did not significantly alter iNOS protein levels.

LPS strongly induced pro-IL-1β protein expression (Figure 8), the precursor form of IL-1β. Although inter-assay variability prevented statistical significance, a trend toward reduced LPS-induced pro-IL-1β expression was observed with fiscalin B derivatives treatment, more pronounced for compounds **2**, **4**, and **7**. None of the compounds alone caused significant changes in iNOS or pro-IL-1β levels when compared to the control.

Analysis of the structure–activity relationship (SAR) reveals that derivatives bearing a *O*-benzylserine substituent (**2**, **3**, and **4**) exhibited the most significant reduction in iNOS levels. This finding suggests that the presence of bulky aromatic moieties at the C-1 position is important for anti-inflammatory activity.

Notably, compounds **2**, **3**, **4**, **6**, and **7** exhibited anti-inflammatory efficacy comparable to that of aprepitant, a known NK_1_R antagonist. At a concentration of 12.5 μM, the pyrazino[2,1-*b*]quinazoline-3,6-dione derivatives reduced NO levels to 41–51% and significantly downregulated iNOS expression. This is comparable to the efficacy of aprepitant, which reduced NO production to approximately 42% at 10 μM in LPS-stimulated RAW264.7 macrophages [57]. Moreover, aprepitant suppressed LPS-induced oxidative stress, attenuated PGE2 production, and downregulated iNOS expression along with pro-inflammatory cytokines. These effects were attributed to the inhibition of the NF-κB signaling pathway [57]. Mechanistically, studies in fibroblast-like synoviocytes demonstrated that aprepitant blocks the TNF-α-induced phosphorylation and degradation of IκBα. This blockade effectively prevents the nuclear translocation of the NF-κB p65 subunit and reduces NF-κB transcriptional activity [58].

**Figure 9:**
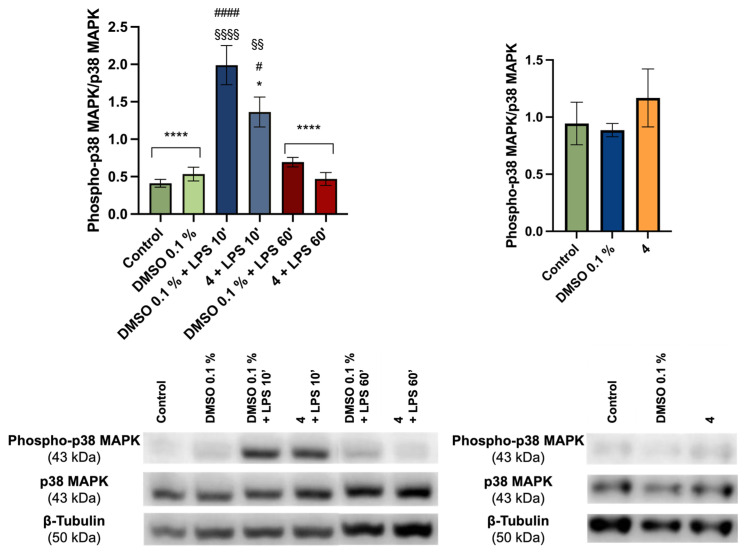
Effect of fiscalin B derivative **4** (12.5 μM) on phospho-p38 MAPK and p38 MAPK levels determined in LPS-activated RAW264.7 cells for 10 and 60 min (**left**), together with the effect of the compounds in non-activated RAW264.7 cells for 60 min (**right**) by Western Blotting. Representative images of the blots are shown; the same loading control was used for phospho-p38 MAPK and p38 MAPK membranes. Data correspond to the mean ± SEM. of at least three independent experiments. Statistical comparisons were made using Ordinary One-way ANOVA followed by Dunnett’s multiple comparisons test (^§§^ *p* < 0.01, ^§§§§^ *p* < 0.0001 vs. DMSO 0.1%; * *p* < 0.05, **** *p* < 0.0001 vs. DMSO 0.1% + LPS 10’; (^#^ *p* < 0.05, ^####^ *p* < 0.0001 vs. DMSO 0.1% + LPS 60’). Legend: Control—untreated cells; DMSO 0.1%—cells treated with 0.1% DMSO; DMSO 0.1% + LPS 10’—LPS-activated cells for 10 min treated with 0.1% DMSO; **4** + LPS 10’—LPS-activated cells for 10’ treated with the fiscalin B derivative **4**; DMSO 0.1% + LPS 60’—LPS-activated cells for 60 min treated with 0.1% DMSO; **4** + LPS 60’—LPS-activated cells for 60’ treated with the fiscalin B derivative **4**.

Since p38 MAPK was identified as a predicted target for fiscalin B derivatives in prior in silico studies, the effect of compound **4** on this specific pathway was evaluated (Figure 9). MAPK activation is a rapid process, with p38 phosphorylation typically peaking within minutes following LPS stimulation, followed by a recovery phase where protein levels return to baseline. This kinetic profile justifies the evaluation of phospho-p38 and total p38 MAPK levels at two critical timepoints: 10 min (peak activation) and 60 min (recovery).

LPS treatment induced a significant increase in phospho-p38 levels at 10 min, returning to near-baseline by 60 min relative to the 0.1% DMSO control. Notably, derivative **4** significantly reduced the phospho-p38/p38 ratio at the 10 min peak relative to DMSO + LPS, with no significant changes detected at 60 min. Additionally, neither 0.1% DMSO alone nor the compound in the absence of LPS elicited significant alterations. This early-phase inhibitory effect on p38 MAPK phosphorylation correlates with the downstream reduction in iNOS and pro-IL-1β, suggesting that attenuation of the p38 MAPK pathway is a primary mechanism of action for this scaffold.

Taken together, these findings suggest that the studied fiscalin B derivatives potentially reduce NO release in LPS-activated macrophages through the significant downregulation of iNOS, with a less pronounced, but indicative effect on pro-IL-1β expression. As previously reported, 17-hydroxybrevianamide N derivatives bearing the pyrazino[2,1-*b*]quinazoline-3,6-dione core exert anti-inflammatory effects via MAPK signaling modulation and NF-κB pathway inhibition [45]. These literature precedents, combined with the observed reduction in p38 phosphorylation, implicate MAPK inhibition as a central mechanism of action for these fiscalin B derivatives.

While studies specifically characterizing the activity of pyrazino[2,1-*b*]quinazoline-3,6-dione derivatives are currently limited, our results provide clear evidence that their anti-inflammatory activity is mediated, at least in part, via the p38-MAPK pathway. Further assays measuring ERK phosphorylation, IκBα degradation, and NF-κB p65 nuclear translocation are necessary to further detail the molecular crosstalk involved.

### 2.7. Structure–Activity Relationship Study Regarding Anti-Inflammatory Properties

**Figure 10:**
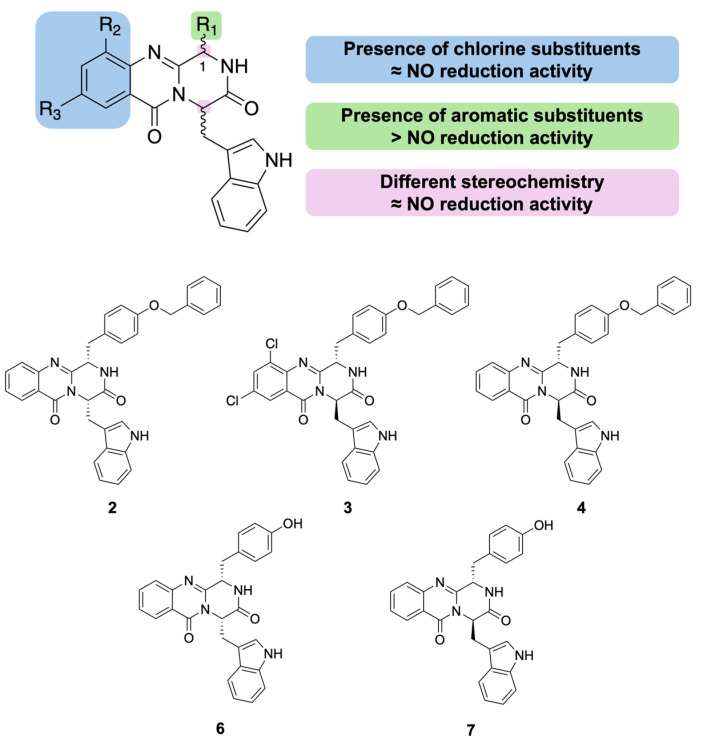
Proposed structure-activity relationship for anti-inflammatory activity and chemical structures of the most promising compounds (**2**, **3**, **4**, **6**, and **7**).

The evaluation of the pyrazino[2,1-*b*]quinazoline-3,6-dione derivatives enabled the identification of preliminary SARs. The most promising compounds, **2**, **3**, **4**, **6**, and **7**, reduced NO production in LPS-activated RAW 264.7 macrophages to levels similar to those of the DMSO 0.1% control, with this effect consistent across all the tested concentrations (12.5–200 μM). All five derivatives share an aromatic substituent at the C-1 position of the scaffold (Figure 10).

Compounds **6** and **7** correspond to the deprotected analogues of **2** and **4**, respectively. While **2** and **6** produced similar NO reductions (48.2% and 51.2% NO production, respectively), **4** showed more pronounced inhibition than **7** (41.3% and 51.1%, difference of approximately 10%). These preliminary data do not allow for definitive conclusions regarding the effect of C-1 aromatic group size, highlighting the need for the synthesis of additional analogues to enable a comprehensive SAR analysis. However, regarding iNOS levels, the *O*-benzylserine substituent (**2**, **3**, and **4**) was associated with a more significant reduction, substantiating the importance of bulkier groups. Compound **3**, a **4** analogue with chlorine substituents on the anthranilic acid ring, exhibited only a modest ~5% decrease in potency relative to **4**, suggesting that these halogens do not affect the observed activity. These findings indicate that: (i) quinazolinone core substitutions do not substantially impact anti-inflammatory activity; (ii) stereochemistry exerts minimal influence; and (iii) the C-1 aromatic substituent is associated with higher reduction of NO production. Figure 9 the SAR preliminary study.

Based on their favorable cytotoxicity profiles in HaCaT and RAW 264.7 cells, along with significant NO reduction, compounds **2**, **3**, **4**, **6**, and **7** were selected for further cytotoxicity and cell migration studies. This selection was also informed by in silico predictions of pharmacokinetics, NK_1_R docking scores, and environmental profiles. Regarding skin permeation, derivatives **2**, **3**, and **4** exhibit higher molecular weights and lipophilicity—potentially suboptimal for dermal delivery due to *stratum corneum* retention—while being predicted as recalcitrant and the most ecotoxic within the series. In contrast, derivatives **6** and **7** display more favorable pharmacokinetic properties for topical absorption and months-scale biodegradability, though aquatic toxicity concerns persist across all candidates. Despite these drawbacks, all five were prioritized due to their superior NK_1_R docking scores over pharmacokinetic and environmental limitations, with future optimizations planned to address these limitations.

### 2.8. Evaluation of Fiscalin B Derivatives on Cytotoxicity and Effect on Cell Migration in NIH/3T3 Cells

**Figure 11:**
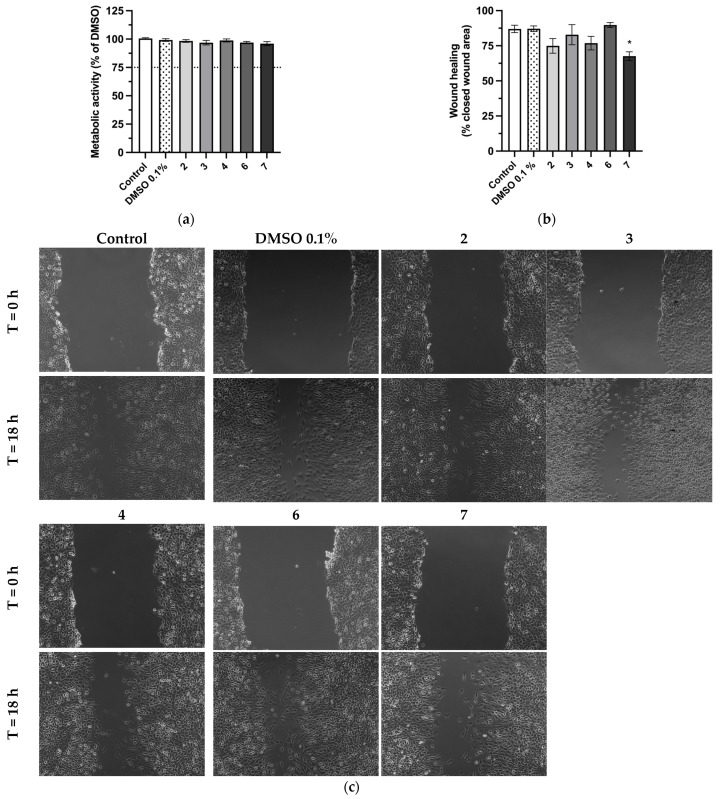
(**a**) Cell metabolic activity of NIH/3T3 cells exposed to 12.5 μM of fiscalin B derivatives **2**, **3**, **4**, **6**, and **7** for 24 h, measured by the Alamar Blue assay; (**b**,**c**) Wound healing effect of the tested compounds on NIH/3T3 fibroblasts. A mechanical scratch was performed, and cells were exposed to the compounds (12.5 μM), vehicle (DMSO 0.1%), or medium alone (Control). Representative microscopy images of NIH/3T3 fibroblasts 0 and 18 h post-scratch are shown in the panel (scale bar = 100 μm). The closure of the wound area was determined by the analysis of the images using the Fiji Is Just ImageJ software. Results are presented as mean ± SEM from three or four independent experiments, performed in duplicate or triplicate. Statistical comparisons were made using Ordinary One-way ANOVA followed by Dunnett’s multiple comparisons test (* *p* < 0.05 vs. DMSO 0.1%).

To support the potential incorporation of fiscalin B derivatives in a pharmaceutical topical product, the cytotoxic profile of the most promising derivatives was evaluated in NIH/3T3 fibroblasts at a concentration of 12.5 μM using the Alamar Blue assay. As depicted in Figure 11a, the derivatives **2**, **3**, **4**, **6**, and **7** demonstrated a safe profile with complete absence of toxicity when compared to the vehicle. Future studies employing primary human dermal fibroblast cultures will be important to further validate the compounds’ biocompatibility and translational relevance for clinical use.

Wound healing is a complex, multi-phase process that includes hemostasis, inflammation, cellular proliferation, and the formation and maturation of granulation tissue. When this process is disrupted, chronic wounds may develop, leading to the persistence of the inflammatory phase [59]. Inflammatory skin disorders accompanied by pruritus, such as atopic dermatitis, are characterized by degradation of the skin barrier, meaning that enhancing tissue regenerative capacity could potentially improve the disorder’s symptoms [60]. Dermal fibroblasts are key mediators of skin repair, contributing to extracellular matrix production, cell migration, re-epithelialization, differentiation, and immune regulation [61]. Taking this into consideration, the migratory capacity of NIH/3T3 fibroblasts was evaluated in the presence of the compounds **2**, **3**, **4**, **6**, and **7** at a concentration of 12.5 μM, or DMSO 0.1% as a vehicle. As shown in Figure 11b,c, none of the tested compounds produced a significant effect on fibroblast migration, except for compound **7,** which significantly inhibited migration. This finding suggests that compound **7** may warrant further investigation in cancer therapy, given its critical role in cell migration, which is essential for cancer invasion and metastasis. In previous screenings, this compound demonstrated a low-to-moderate cytotoxic effect against a panel of human tumor cell lines (NCI-H460, BxPC3, and PANC1), displaying 50% growth inhibition (GI_50_) concentrations ranging from 34.13 to 50.59 μM [62], which supports the observed inhibition. Although no increase in regenerative potential was observed relative to the other compounds, the preservation of basal regenerative capacity is a favorable outcome.

## 3. Materials and Methods

### 3.1. Docking Studies

The three-dimensional crystal structure of the human NK_1_R (PDB ID: 6E59) was retrieved from the Protein Data Bank (PDB). The structure was determined by X-ray diffraction at a resolution of 3.40 Å. The target protein was prepared for docking using AutoDockTools (ADT, The Scripps Research, La Jolla, CA, USA) following these steps: (i) removal of all non-essential heteroatoms, including water molecules and the co-crystallized ligand; (ii) addition of all polar hydrogen atoms; (iii) assignment of Kollman united atom partial charges and conversion of the receptor to the PDBQT file format required by AutoDock Vina.

The following established NK_1_R antagonists were used as control compounds: aprepitant, rolapitant, serlopitant, netupitant, tradipitant, and CP-99,995. All compounds’ structures were initially drawn using ChemDraw Professional 16.0 (Perkin Elmer Informatics, Waltham, MA, USA). Conformational energy minimization was performed using ArgusLab 4.0.1 software (Mark Thompson and Planaria Software LLC, Richland, WA, USA). Minimization utilized the semi-empirical quantum mechanical (QM) method under the Hamiltonian setting. Specifically, the Austin Model 1 (AM1) parameterization was selected, or the Parametric Method 3 (PM3) method was chosen when halogens were present in the ligand structure. The calculation proceeded until the energy gradient between consecutive steps in the geometry search was inferior to 0.1 kcal. Å^−1^·mol^−1^. Partial atomic charges were subsequently calculated following the standard parameters of the force field used. The stereochemistry of each compound was preserved throughout the process. Finally, the optimized ligand structures were converted to the PDBQT format using ADT. Molecular docking was performed using AutoDock Vina v1.1.2. (The Scripps Research, La Jolla, CA, USA) [29]. The docking process was managed through the graphical user interface PyRx v0.8 (The Scripps Research Institute, La Jolla, CA, USA). The docking procedure considered the target receptor as rigid and the ligands as flexible. The search space (grid box) was centered over the known orthosteric binding pocket, defined by visually identifying the center of the co-crystallized ligand in the NK_1_R structure. The final parameters defined in PyRx were center: X: −17.65 Å, Y: 54.54 Å, Z: 55.62 Å, and dimensions: X: 19.00 Å, Y: 21.20 Å, Z: 22.61 Å. The exhaustiveness parameter, which controls the thoroughness of the search, was set to 8. Results were ranked based on the predicted binding affinity, expressed as the docking score (kcal·mol^−1^). For molecular visualization, PyMol v3.1 (Schrödinger, New York, NY, USA) was used [63].

To validate the docking protocol, a re-docking procedure was performed using the co-crystallized ligand from the structure (PDB ID: 6E59). The ligand was extracted and re-docked into the defined binding site using the same parameters applied to the fiscalin B derivatives. The protocol’s reliability was assessed by calculating the Root Mean Square Deviation (RMSD) between the predicted docking pose and the experimental crystallographic orientation. The resulting RMSD was 0.303 Å, confirming that the selected docking parameters are robust and capable of accurately predicting the binding orientations of the study compounds.

### 3.2. In Silico Studies

The dermatopharmacokinetic properties of fiscalin B derivatives were predicted in silico using the free web-based online platforms SwissADME [64] and ADMETlab 3.0 [65]. The chemical structures were entered in SMILES format, generated using ChemDraw Professional v16.0. The calculated parameters comprised molecular weight, hydrophobicity (Log P), Log Kp, and the probability of skin sensitization and eye irritation.

The biodegradability and the aquatic toxicity of the fiscalin B derivatives were predicted with the software EPI Suite^TM^ v4.11 from U.S. Environmental Protection Agency (Washington, DC, USA) [30]. The biodegradability was evaluated using the module BIOWIN v4.10 with base in criteria for easy biodegrading. The chemical’s acute and chronic aquatic toxicity was estimated with ECOSAR v1.11, a module that predicts the mean lethal concentration (50%, LC_50_) and mean effective concentration (50%, EC_50_) for fishes, invertebrates (*Daphnia*) and algae, together with chronic values (ChV), based on SAR. All the molecular structures were introduced in the SMILES format generated using the ChemDraw Professional v16.0 software. Compounds were grouped into four structural classes (amides, pyrazoles/pyrroles, phenols, and phenol amines) to evaluate the potential risk for aquatic species, the obtained acute toxicity was compared to categories of concern for aquatic species defined by the U.S. EPA based on the acute threshold as follows: very highly toxic (≤1 mg·L^−1^), moderately toxic (>1 to ≤10 mg·L^−1^), slightly toxic (>10 to ≤100 mg·L^−1^), and practically nontoxic (>100 mg·L^−1^) [66].

Using SwissTargetPrediction, a free web-based online platform [32], potential targets for the derivatives were predicted. The chemical structures were input in SMILES format, generated using ChemDraw Professional v16.0 software and the selected species was *Homo sapiens*. For each derivative, the 25 most probable protein targets were ranked, identifying a total of 192 targets across various therapeutic classes. Since the 25 targets obtained for each compound exhibited the same probability, ranging from 7% to 12% across the series, they could not be ranked by score. Therefore, the selection was based on prediction frequency, with the 25 most frequently predicted targets identified and listed.

### 3.3. Tested Compounds

The synthesis of the following compounds was previously described: Fiscalin B, **9**, **12**, **13** [19]; **2**, **4**, **6**, **7**, **14** [18]; **3**, **8**, **10**, **11**, **15**, **16**, **17** [22]; **1**, **5** [67]. A 50 mM stock solution of each compound was prepared in DMSO, filtered by using a 0.22 µm sterile filter and stored at −20 °C. Serial dilutions were made with medium to obtain the final concentrations of 12.5, 25, 50, 100, and 200 μM in the wells.

### 3.4. Cell Culture

The murine macrophage cell line RAW 264.7 (ATCC No. TIB-71, Manassas, VA, USA) was used as a cellular model of inflammation and was cultured in Dulbecco’s Modified Eagle Medium (DMEM, ThermoFisher Scientific, Walthman, MA, USA) supplemented with 10% (*v*/*v*) non-inactivated fetal bovine serum (FBS, ThermoFisher Scientific), 100 U·mL^−1^ penicillin (Sigma-Aldrich Co., St Louis, MO, USA), 100 µg·mL^−1^ streptomycin (Sigma-Aldrich Co.), 1.5 g·L^−1^ sodium bicarbonate, and 1 mM sodium pyruvate (Sigma-Aldrich Co.). The cell line was maintained and incubated at 37 °C in an atmosphere of 5% CO_2_. When 70–80% confluence was reached, cells were mechanically detached for passage and/or seeding.

The human keratinocytes (HaCaT, CLS 300493, Eppelheim, Germany) cell line and NIH/3T3 (embryonic mouse fibroblast cell line, ATCC CRL-1658, Manassas, Virginia) cell line were cultured with DMEM (ThermoFisher Scientific) supplemented with 10% (*v*/*v*) heat-inactivated FBS (ThermoFisher Scientific), 100 U·mL^−1^ penicillin (Sigma-Aldrich Co.), 100 µg streptomycin (Sigma-Aldrich Co.), 3.7 g·L^−1^ sodium bicarbonate, and 1 mM sodium pyruvate (Sigma-Aldrich Co.). The cells were cultured in 75 cm^2^ flasks and maintained in a humidified 5% CO_2_ atmosphere at 37 °C, with medium changed every 2–3 days. Upon reaching 70–80% confluence, cells were washed with a phosphate-buffered saline (PBS) with 0.05% ethylenediamine tetraacetic acid (EDTA, Sigma-Aldrich Co.), followed by detachment with 1× Trypsin-EDTA solution (Sigma-Aldrich Co.), for passage or seeding. For cell viability assessment, cells were seeded in 96-well plates at a density of 2.0 × 10^4^ cell·mL^−1^ and 1 × 10^4^ cells·mL^−1^ for HaCaT and NIH/3T3 cells, respectively, and allowed to adhere for 24 h. In the case of NIH/3T3 cells, the cell culture medium was replaced with freshly prepared DMEM supplemented with only 2% (*v*/*v*) FBS.

### 3.5. Cellular Metabolic Activity Measurement

To evaluate the metabolic activity and, consequently, cell viability, the colorimetric Alamar Blue assay, also known as resazurin reduction assay, was used. Viable, metabolically active cells reduce resazurin to the fluorescent product pink colored product, resorufin, that can be determined fluorometrically [68]. Cells were plated in a 96-well plate and stabilized overnight, at a cellular density of 5 × 10^4^ cells/well, 1.0 × 10^5^ cell/well and 1 × 10^4^ cells/well for RAW 264.7, HaCaT, and NIH/3T3 cells, respectively. Initial seeding densities were adjusted according to the specific growth characteristics of each cell type to avoid contact inhibition and metabolic exhaustion during the treatment period. On the experiment day, the medium was replaced by freshly prepared DMEM supplemented with 10% FBS, in the case of RAW 264.7 and HaCaT cell lines, and with 1% FBS in the case of NIH/3T3 cells. Then, cells were exposed to different concentrations of the compounds and incubated at 37 °C in an atmosphere of 5% CO_2_. After 24 h, a 50 μM resazurin solution (Sigma-Aldrich Co.), prepared in PBS, was added to RAW 264.7 cells and incubated for 1.5 h. For HaCaT and NIH/3T3 cell lines, the 50 μM resazurin solution was added 20 h after exposure to the compounds and incubated for an additional 4 h. The absorbance was measured at wavelengths of 570 and 620 nm using a microplate reader from BioTek Synergy HT (BioTek, Winooski, VT, USA). The compound concentrations that induced less than a 25% decrease in fluorescence were chosen for the subsequent assays. All the experiments were performed in duplicate or triplicate, with a minimum of three independent assays being graphically represented as the percentage of cell viability versus the concentrations of the tested compounds compared with the control.

### 3.6. Anti-Inflammatory Activity Evaluation in RAW264.7 Macrophage Cells

LPS was used to stimulate the production of the inflammatory mediator NO. The ability of the tested compounds to decrease the NO production was evaluated spectrophotometrically by measuring the amount of an oxidative product of NO in cell supernatants using the Griess assay [69]. After being seeded and stabilized overnight, cells were pre-exposed for one hour to different non-cytotoxic concentrations of the compounds. A solution of LPS (50 ng/mL in PBS, Sigma-Aldrich Co.) was added, and after 24 h, an equal volume of cell supernatant and Griess reagent ([1:1] mixture of 1% (*w*/*v*) sulfanilamide (Sigma-Aldrich Co.) in 5% (*v*/*v*) phosphoric acid (Alfa Aesar, Ward Hill, MA, USA), and 0.1% (*w*/*v*) *N*-(1-naphthyl)-ethylenediamine dihydrochloride (Sigma-Aldrich Co.) was added. The mixture was further incubated for 30 min, protected from light. The absorbance was measured at 550 nm using a microplate reader from BioTek Synergy HT (BioTek), and the nitrite concentration accumulated in the supernatants was extrapolated from a sodium nitrate standard curve. All the experiments were performed in duplicate with a minimum of three independent assays. The results are expressed as the percentage of NO production by cells treated with LPS and the compounds, compared to LPS alone (100%).

### 3.7. Nitric Oxide Scavenging Assay

The compounds’ NO scavenging potential was evaluated in chimico using SNAP as NO donor and the Griess assay to evaluate NO production, as previously reported [70]. In 48-well plates, 300 μL of medium or medium containing the compound dissolved in DMSO, along with 300 μM of SNAP, were incubated at 37 °C. After three hours, the Griess assay was performed, where equal proportions of the supernatants and Griess reagent [1:1 of 1% (*w/v*) sulphanilamide in 5% (*v/v*) phosphoric acid and 0.1% (*w/v*) *N*-(1-naphthyl)ethylenediamine dihydrochloride] were mixed and incubated in the dark for 30 min at room temperature. The absorbance was measured at 550 nm using a Biotek Synergy HT plate reader (Biotek, El Segundo, CA, USA). The accumulated nitrite concentration in supernatants was calculated by interpolation of the absorbance in a standard curve of sodium nitrite. Three independent assays were performed in duplicate.

### 3.8. Protein Extraction and Western Blot

RAW 264.7 cells were seeded into a 12-well plate at a density of 2.5 × 10^4^ cells·mL^−1^ and allowed to adhere overnight. Cells were subsequently pre-treated for one hour with non-cytotoxic concentrations of the test compounds, previously determined to inhibit NO production. Following pre-treatment, cells were stimulated with LPS at 50 ng·mL^−1^ for 24 h. To prepare total cellular extracts, the culture medium was aspirated, and the cells were washed with ice-cold PBS. Cells were detached mechanically using a cell scraper, collected, and centrifuged at 500× *g* for 5 min at 4 °C. The resulting cell pellet was lysed in 100 μL of RIPA lysis buffer [150 mM NaCl (ThermoFisher Scientific), 50 mM Tris-HCl (pH 8.0) (Stratagene California, La Jolla, CA, USA), 2 mM EDTA (Sigma-Aldrich), 0.5% (*w*/*v*) sodium deoxycholate (Sigma-Aldrich), 0.1% (*w*/*v*) sodium dodecyl sulphate (SDS, Sigma-Aldrich), and 1% Nonidet P-40 (Sigma-Aldrich)]. The lysis buffer was supplemented with 1 mM dithiothreitol (DTT, PanReac AppliChem, Barcelona, Spain) and a phosphatase (1:10) and protease (1:7) inhibitor cocktail (Roche Diagnostics, Mannheim, Germany). Lysis was performed on ice for 30 min. The lysates were then centrifugated at 12,000× *g* for 10 min at 4 °C, and the resulting supernatant was collected and stored at −80 °C until further analysis.

The total protein concentration of the cell lysates was determined using the Bicinchoninic Acid assay kit (Sigma-Aldrich). The lysates were denatured by mixing with a 2× concentrated denaturing solution [0.25 M Tris (pH 6.8), 4% (*w*/*v*) SDS, 200 mM DTT, 20% (*v*/*v*) glycerol (Sigma-Aldrich), and bromophenol blue] for 5 min at 95 °C. For the determination of specific target proteins, 30 μg of total protein per sample were resolved by 10% (*v*/*v*) SDS-Polyacrylamide Gel Electrophoresis (SDS-PAGE). The separated proteins were then electrotransferred onto polyvinylidene difluoride (PVDF) membranes (Millipore Corporation, Burlington, MA, USA). Membranes were blocked for one hour at room temperature with a solution of 5% (*w*/*v*) non-fat dry milk prepared in tris-buffered saline with tween 20 (TBS-T) (150 mM NaCl, 25 mM Tris-HCl at pH 7.0, and 0.1% Tween 20). The membranes were subsequently incubated overnight at 4 °C with the primary antibodies against iNOS (1:1000) (MAD9502, R&D Systems, Minneapolis, MN, USA), pro-IL-1β (1:500) (#ab9722, Abcam, Cambridge, UK), and β-tubulin (1:20,000) (#T7816, Sigma-Aldrich Chemical, St. Louis, MO, USA). Following three washes (10 min each) with TBS-T, the membranes were incubated for one hour at room temperature with the respective horseradish peroxidase-conjugated secondary antibodies (anti-rabbit or anti-mouse), both diluted 1:2000 (#7074 and #7076, respectively, Cell Signaling Technology, Danvers, MA, USA). Blots were visualized using the enhanced chemiluminescence method with the ImageQuant TM LAS 500 (GE, Healthcare, Chicago, IL, USA) and analyzed quantitatively using Total Lab (version 2009) software.

### 3.9. Wound Healing Effect

Mouse fibroblasts (NIH/3T3, ATCC CRL-1658, Manassas, VA, USA) were cultured in DMEM (Sigma-Aldrich) supplemented with 10% (*v*/*v*) heat-inactivated FBS, 1% (*v/v*) antibiotic solution (from a stock solution with 10,000 U·mL^−1^ penicillin and 10,000 µg·mL^−1^ streptomycin; Gibco, Carlsbad, CA, USA), 25 mM of glucose, and 3.7 g·L^−1^ of sodium bicarbonate. Cells were cultured and maintained in 75 cm^2^ flasks in a humidified 5% CO_2_-95% air atmosphere at 37 °C, with changes of medium every 2–3 days. Upon reaching 70–80% confluence, cells were detached with Trypsin-EDTA solution 1× (Sigma-Aldrich).

To evaluate the effect of compounds on cell migration, the scratch wound assay described by Martinotti et al. [71] was selected. Cells were seeded into 12-well plates at a density of 250,000 cells·mL^−1^. After approximately 24 h, to allow cells to reach about 95% of confluence, a vertical scratch was made across the cell monolayer using a P10 pipette tip. Eight photographs were captured immediately after scratching (T0 = 0 h) using a Zeiss Axio HXP IRE 2 microscope equipped with an EC plan-Neofluar 10× objective (Carl Zeiss, Oberkochen, Germany). Cells were then exposed to cell culture medium alone, with vehicle, or with the compounds under test (12.5 μM), all containing 2% (*v*/*v*) FBS heat-inactivated to reduce cell proliferation rate during the assay. After 18 h (T18), photographs at the same coordinates as in T0 were recaptured. The open wound area was quantified using the ImageJ/FIJI plugin v2.16.0 [72] (variance window radius -10; threshold value—100; percentage of saturated pixels—0.001). The migration rate of four independent assays performed in duplicate was calculated using the following equation:

(1) $$\%closedwoundarea=\frac{{T0}_{cellfreearea}-{T18}_{cellfreearea}}{{T0}_{cellfreearea}}\times 100$$

where T0_cell free area_ corresponds to the measured area at the time point zero, immediately after performing the scratch and T18_cell free area_ corresponds to the measured area 18 h after performing the scratch.

## 4. Conclusions

Inspired by the marine natural product SP antagonist fiscalin B, synthetized pyrazino[1,2-*b*]quinazoline-3,6-dione derivatives emerged from a docking-based virtual screening as promising NK_1_R high-affinity ligands with predicted binding affinities comparable to, or exceeding, the reference drug aprepitant. Hydrophobic substituents placed in pyrazino[1,2-*b*]quinazoline-3,6-dione moiety, particularly aromatic substituents at position 1, appear to be associated with a higher docking score and highlighted **1**, **2**, **3**, **4**, and **5** as hit compounds for further computational and experimental investigations.

In silico dermatopharmacokinetic and ecotoxicological properties evaluation revealed that several derivatives fulfil basic requirements for topical delivery, but many display high lipophilicity, poor biodegradability, and relevant predicted aquatic toxicity, especially halogenated and benzyl-protected analogues, underscoring the need to balance pharmacological potency with environmental and safety considerations. Target prediction supported a polypharmacological profile converging on inflammatory nodes such as MAPKs, cathepsins, nitric oxide synthases, growth factor receptors, and epigenetic regulators, suggesting that the derivatives may exert multi-target anti-inflammatory activities.

In LPS-stimulated RAW 264.7 macrophages, most fiscalin B derivatives reduced NO production at non-cytotoxic concentrations, with **2**, **3**, **4**, **6**, and **7** showing robust, concentration-independent inhibition. NO scavenging was not detected in the SNAP assay, while derivatives **2**, **3**, and **4** significantly reduced iNOS levels, pointing to modulation of inflammatory signaling rather than direct radical quenching. Mechanistic evaluation of the predicted MAPK target confirmed that derivative **4** significantly attenuates the LPS-induced phosphorylation of p38 MAPK during its peak activation phase. This early-phase inhibition of the p38 pathway correlates with the observed reduction in iNOS and pro-IL-1β, identifying MAPK modulation as a primary mechanism of action. In HaCaT keratinocytes and NIH/3T3 fibroblasts, these derivatives showed no cytotoxicity at 12.5 µM and did not affect cell migration, except for **7**, which inhibited migration, highlighting its potential relevance for oncology applications. Notably, our data indicated that aromatic substituents at the C-1 position of the pyrazino[2,1-*b*]quinazoline-3,6-dione derivatives core enhance both in vitro anti-inflammatory and predicted NK_1_R binding affinity, suggesting that further exploration of π-stacking-enhancing groups in this region could yield optimized derivatives with potential dual-functionality. Moreover, both predicted NK_1_R affinity and the observed anti-inflammatory efficacy appear independent of stereochemistry, suggesting that the configurations at C-1 and C-4 do not significantly affect these activities.

In addition, experimental validation of the in silico predictions is required, particularly through NK_1_R binding and target-based assays to confirm the computationally predicted affinities and anti-inflammatory mechanisms. Moving forward, considering the data reported in this work, research should prioritize the systematic characterization of NK_1_R interactions alongside the rational design of molecular modifications—namely the reduction in lipophilicity and molecular weight—aiming to enhance biodegradability and skin permeation, and reduce aquatic toxicity, while retaining both anti-inflammatory efficacy and predicted NK_1_R affinity. Subsequent studies should validate the most promising candidates in sophisticated in vitro three-dimensional skin models and in vivo inflammatory models, incorporating comprehensive evaluations of skin permeation, sensitization potential, and environmental fate, thereby bridging molecular design with translational safety and efficacy.

## Figures and Tables

**Table 1:** Docking scores of the fiscalin B derivatives, together with fiscalin B and aprepitant, for the neurokinin 1 receptor (NK_1_R) binding pocket, calculated using Autodock Vina.

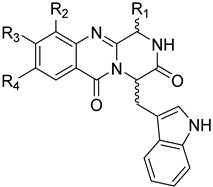						
Compound	Stereochemistry	Substituents/Chemical Structure				Docking Score (kcal·mol^−1^)
		R_1_	R_2_	R_3_	R_4_	
**1**	(1*S*,4*R*)	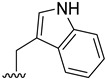	H	H	H	−11.9
**2**	(1S,4S)	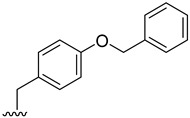	H	H	H	−11.3
**3**	(1*S*,4*R*)	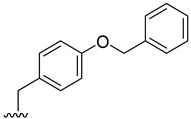	Cl	H	Cl	−11.2
**4**	(1*S*,4*R*)	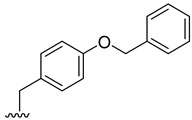	H	H	H	−11.1
**5**	(1*S*,4*R*)	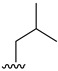	H	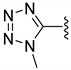	H	−10.9
**6**	(1S,4S)	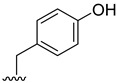	H	H	H	−10.8
**7**	(1*S*,4*R*)	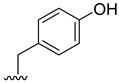	H	H	H	−10.7
**8**	(1*S*,4*R*)	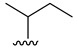	Cl	H	Cl	−10.3
**9**	(1*R*,4*S*)	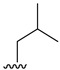	H	H	H	−10.3
**10**	(1*S*,4*R*)	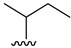	H	H	Cl	−10.2
**11**	(1*S*,4*R*)	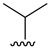	Cl	H	Cl	−10.2
**12**	(1*S*,4*S*)	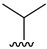	H	H	H	−10.2
**13**	(1*R*,4*S*)	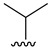	H	H	H	−10.2
**14**	(1*R*,4*R*)	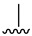	H	H	H	−10.2
**15**	(1*S*,4*R*)	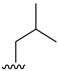	H	H	Br	−10.2
**16**	(1*S*,4*R*)	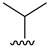	H	H	Cl	−10.1
**17**	(1*S*,4*R*)	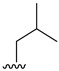	H	H	I	−10.1
**Fiscalin B**	(1*S*,4*R*)	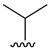	H	H	H	−10.1
**Aprepitant**	-	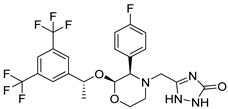				−10.9

## Data Availability

The original contributions presented in this study are included in the article/Supplementary Material. Further inquiries can be directed to the corresponding author.

## Supplementary Materials

The following supporting information can be downloaded at: https://www.mdpi.com/article/10.3390/ph19050775/s1, Table S1: Summary of key amino acid residues in the neurokinin 1 receptor (NK1R) binding site that establish interactions with fiscalin B, its derivatives, and aprepitant; Table S2: In silico prediction of the important ADMET properties for topical application; Table S3: Complete prediction of biodegradability and aquatic toxicity of fiscalin B and its derivatives, together with aprepitant performed in silico using the ECOSAR software v1.11; Table S4: Summary of the data obtained by ECOSAR for the aprepitant, fiscalin B, and its derivatives; Figure S1: Cell metabolic activity of HaCaT cells exposed to increasing concentrations of fiscalin B and its derivatives (0–200 μM) for 24 h, measured by the Alamar Blue assay. Values are the mean ± SEM of at least four independent experiments performed in duplicate. Statistical comparisons were made using Ordinary One-way ANOVA followed by Dunnett’s multiple comparisons test (* *p* < 0.05, ** *p* < 0.01, *** *p* < 0.001, **** *p* < 0.0001 vs. DMSO 0.1%); Figure S2: Cell metabolic activity of RAW 264.7 cells exposed to increasing concentrations of fiscalin B and its derivatives (0–200 μM) alone or followed by LPS (100 ng·mL^−1^), for 24 h, measured by the Alamar Blue assay. Values are the mean ± SEM of at least four independent experiments performed in duplicate. Statistical comparisons were made using Ordinary One-way ANOVA followed by Dunnett’s multiple comparisons test (* *p* < 0.05, ** *p* < 0.01, *** *p* < 0.001, **** *p* < 0.0001 vs. DMSO 0.1%); Figure S3: Effect of fiscalin B and derivatives on NO production. Nitrite levels were determined using the Griess assay in RAW 264.7 cells exposed to fiscalin derivatives (12.5–200 μM) in the absence or in the presence of LPS for 24 h. Values are the mean ± SEM of at least three independent experiments performed in duplicate. Statistical comparisons were made using Ordinary One-way ANOVA followed by Dunnett’s multiple comparisons test (**** *p* < 0.0001 vs. DMSO 0.1% + LPS; ^#^ *p* < 0.05, ^##^ *p* < 0.01, ^###^ *p* < 0.001, ^####^ *p* < 0.0001 vs. DMSO 0.1%).

## Abbreviations

The following abbreviations are used in this manuscript:

NK_1_R	Neurokinin-1 receptor
SP	Substance P
NF-κB	Nuclear factor kappa B
MAPK	Mitogen-activated protein kinase
LPS	Lipopolysaccharide
SNAP	S-nitroso-N-acetyl-D,L-penicillamine
NO	Nitric oxide
iNOS	Inducible nitric oxide synthase
pro-IL-1β	Pro-interleukin-1 beta
SAR	Structure–activity relationship
