# Supplementary material for: Anti-Inflammatory Evaluation of Pyrazino[2,1-b]quinazoline-3,6-dione Derivatives Inspired by Fiscalin B
Source: Pharmaceuticals (Basel). 2026 May 15;19(5):775. doi: 10.3390/ph19050775 (PMC13210532; doi:10.3390/ph19050775)
Supplement: Supplementary file 1 [file pharmaceuticals-19-00775-s001.zip › pharmaceuticals-4302914-supplementary.pdf]

Supporting information

Anti-Inflammatory Evaluation of Pyrazino[2,1-*b*]quinazoline-3,6-dione Derivatives Inspired by Fiscalin B

Márcia S. Martins[1,2], Madalena M. M. Pinto[1,2], Isabel F. Almeida[3,4], Maria T. Cruz[5,6], Emília Sousa[1,2*]

1 Laboratório de Química Orgânica e Farmacêutica, Departamento de Ciências Químicas, Faculdade de Farmácia, Universidade do Porto, 4050-313 Porto, Portugal; up201605865@ff.up.pt (M.S.M.); madalena@ff.up.pt (M.M.M.P.); esousa@ff.up.pt (E.S.)
2 CIIMAR—Centro Interdisciplinar de Investigação Marinha e Ambiental, 4450-208 Matosinhos, Portugal;
3 UCIBIO, i4HB, Faculdade de Farmácia, Universidade do Porto, Rua de Jorge Viterbo Ferreira, 228, 4050-313 Porto, Portugal; ifalmeida@ff.up.pt
4 Laboratory of Pharmaceutical Technology, Faculty of Pharmacy, University of Porto, 4050-313 Porto, Portugal;
5 Faculty of Pharmacy, University of Coimbra, 3004-531 Coimbra, Portugal; trosete@ff.uc.pt
6 CNC—Center for Neuroscience and Cell Biology, University of Coimbra, 3004-504 Coimbra, Portugal.
* Correspondence: esousa@ff.up.pt

Table S1. Summary of key amino acid residues in the neurokinin 1 receptor (NK_1R) binding site that establish interactions with fiscalin B, its derivatives, and aprepitant.

Code	H-bond interactions	Non-polar interactions
**1**	Ile182	Phe90, Ile113, Val116, Tyr192, Tyr196, His197, Val200, Ile204, Trp261, Phe268
**2**	Gln165, Thr201	Phe90, Ala93, Trp98, Ile113, Ile182, Val200, Ile204, Phe264, Phe268, Leu269, Tyr287
**3**	Tyr272	Phe90, Ile113, Val200, Trp261, Phe264, Phe267, Phe268
**4**	Gln165 (double interaction)	Phe90, Ile113, Tyr196, Val200, Ile204, Phe264, Phe267, Phe268, Tyr272, Tyr287
**5**	His108, Asn109, Tyr272	Ile113, Val116, Tyr196, Val200, Trp261, Phe264, Phe268, Tyr287
**Fiscalin B**	Thr201	Phe90, Ile113, Val116, Tyr196, Val200, Ile204, Trp261, Phe268

**Aprepitant**	Glu193, Tyr272	Phe90, Ile113, Val116, Tyr196, Val200, Ile204, Trp261, Phe264, Phe268

The table categorizes hydrogen bond (H-bond) and non-polar (hydrophobic) interactions (defined by residues located within 4 Å of the ligand). The designation "double interaction" indicates that the ligand forms two distinct hydrogen bonds with the same amino acid residue. Residues involved in the interactions are labeled: TYR-tyrosine; PHE-phenylalanine; TRP-tryptophan; VAL-valine; ILE-isoleucine; GLU-glutamic acid; THR-threonine; GLN-glutamine; ALA-alanine; LEU-leucine.

Table S2. *In silico* prediction of the important ADMET properties for topical application.

Compound	Molecular Weight	Log P	log Kp (cm.s^{-1})	Skin Sensitization (%)	Eye irritation (%)
**1**	473.53	3.06	-6.16	22.7	1.7
**2**	540.61	4.32	-5.62	2.2	2.5
**3**	609.50	5.39	-5.15	17.9	0.2
**4**	540.61	4.35	-5.62	2.5	3.4
**5**	482.54	2.86	-6.99	5.2	3.6
**6**	450.49	2.75	-6.36	8.8	12.4
**7**	450.49	2.74	-6.36	14.9	26.4
**8**	469.36	4.22	-5.53	66.1	17.7
**9**	400.47	3.47	-6.00	62.1	14.3
**10**	434.92	3.96	-5.77	37.5	23.3
**11**	455.34	3.89	-5.70	79.8	13.0
**12**	386.45	2.64	-6.17	83.6	23.6
**13**	386.45	2.73	-6.17	69.2	8.5
**14**	358.39	2.17	-6.68	29.5	16.8
**15**	479.37	4.10	-5.99	44.8	41.4
**16**	420.89	3.62	-5.93	55.6	20.2
**17**	526.37	4.23	-6.30	53.2	23.2
**Fiscalin B**	386.45	2.87	-6.17	56.8	43.8
**Aprepitant**	534.43	3.80	-6.58	0.1	20.5

Log P - octanol-water partition coefficient, Log Kp - skin permeation coefficient

Table S3. Complete prediction of biodegradability and aquatic toxicity of fiscalin B and its derivatives, together with aprepitant performed *in silico* using the ECOSAR software v1.11.

Compound	Biodegradability	Aquatic toxicity							
		ECOSAR Class	Organism	Duration	END PT	Predicted ($mg.mL^{-1}$)	Organism	END PT	Predicted ($mg.mL^{-1}$)
Aprepitant	Not readily biodegradable; recalcitrant	Aliphatic Amines	Fish	96-hr	LC50	3.849*	Fish	ChV	0.104
			Daphnid	48-hr	LC50	0.591*	Daphnid	ChV	0.062
			Green Algae	96-hr	EC50	0.296*	Green Algae	ChV	0.118
		Hydrazines	Fish	96-hr	LC50	0.856*	Fish	ChV	0.025
			Daphnid	48-hr	LC50	2.275*	Daphnid	ChV	0.102
			Green Algae	96-hr	EC50	0.283*	Green Algae	ChV	0.115
Fiscalin B	Not readily biodegradable; biodegradable in months	Amides	Fish	96-hr	LC50	87.985*	Fish	ChV	1.531
			Daphnid	48-hr	LC50	89.783*	Daphnid	ChV	0.00154
			Green Algae	96-hr	EC50	2.867	Green Algae	ChV	2.763
			Fish (SW)	96-hr	LC50	77.846*	-	-	-
			Mysid (SW)	96-hr	LC50	5.186	-	-	-
		Pyrazoles/Pyrroles	Fish	96-hr	LC50	6.184	Fish	ChV	0.166
			Daphnid	48-hr	LC50	9.544	Daphnid	ChV	5.616
			Green Algae	96-hr	EC50	10.824	Green Algae	ChV	2.887
1	Not readily biodegradable; biodegradable in months	Amides	Fish	96-hr	LC50	26.124*	Fish	ChV	0.082
			Daphnid	48-hr	LC50	17.929*	Daphnid	ChV	1.980*
			Green Algae	96-hr	EC50	1.025*	Green Algae	ChV	1.484*
			Fish (SW)	96-hr	LC50	23.316*	-	-	-
			Mysid (SW)	96-hr	LC50	2.038*	-	-	-
		Pyrazoles/Pyrroles	Fish	96-hr	LC50	1.324*	Fish	ChV	0.576*
			Daphnid	48-hr	LC50	7.842*	Daphnid	ChV	0.00173
			Green Algae	96-hr	EC50	3.182*	Green Algae	ChV	1.216*
2		Amides	Fish	96-hr	LC50	1.736*	Fish	ChV	0.015
			Daphnid	48-hr	LC50	0.538*	Daphnid	ChV	0.186*

	Not readily biodegradable; recalcitrant		Green Algae	96-hr	EC50	0.099*	Green Algae	ChV	0.297*
			Fish (SW)	96-hr	LC50	1.577*	-	-	-
			Mysid (SW)	96-hr	LC50	0.238*	-	-	-
		Pyrazoles/Pyrroles	Fish	96-hr	LC50	0.046*	Fish	ChV	0.061*
			Daphnid	48-hr	LC50	4.016*	Daphnid	ChV	0.00165
			Green Algae	96-hr	EC50	0.207*	Green Algae	ChV	0.178*
3	Not readily biodegradable; recalcitrant	Amides	Fish	96-hr	LC50	0.234*	Fish	ChV	0.004*
			Daphnid	48-hr	LC50	0.040*	Daphnid	ChV	0.032*
			Green Algae	96-hr	EC50	0.018*	Green Algae	ChV	0.091*
			Fish (SW)	96-hr	LC50	0.216*	-	-	-
			Mysid (SW)	96-hr	LC50	0.049*	-	-	-
		Pyrazoles/Pyrroles	Fish	96-hr	LC50	0.004*	Fish	ChV	0.012*
			Daphnid	48-hr	LC50	2.489*	Daphnid	ChV	0.00163*
			Green Algae	96-hr	EC50	0.028*	Green Algae	ChV	0.043*
4	Not readily biodegradable; recalcitrant	Amides	Fish	96-hr	LC50	1.736*	Fish	ChV	0.015
			Daphnid	48-hr	LC50	0.538*	Daphnid	ChV	0.297*
			Green Algae	96-hr	EC50	0.099*	Green Algae	ChV	0.297*
			Fish (SW)	96-hr	LC50	1.577*	-	-	-
			Mysid (SW)	96-hr	LC50	0.238*	-	-	-
		Pyrazoles/Pyrroles	Fish	96-hr	LC50	0.046*	Fish	ChV	0.061*
			Daphnid	48-hr	LC50	4.016*	Daphnid	ChV	0.00165
			Green Algae	96-hr	EC50	0.207*	Green Algae	ChV	0.178*
5	Not readily biodegradable; biodegradable in months	Amides	Fish	96-hr	LC50	391.916*	Fish	ChV	0.468
			Daphnid	48-hr	LC50	570.866*	Daphnid	ChV	21.443
			Green Algae	96-hr	EC50	10.811	Green Algae	ChV	7.860
			Fish (SW)	96-hr	LC50	344.049*	-	-	-
			Mysid (SW)	96-hr	LC50	17.964	-	-	-
		Pyrazoles/Pyrroles	Fish	96-hr	LC50	36.936	Fish	ChV	5.518
			Daphnid	48-hr	LC50	17.057	Daphnid	ChV	0.002
			Green Algae	96-hr	EC50	48.644	Green Algae	ChV	8.647

6	Not readily biodegradable; biodegradable in months	Phenols	Fish	96-hr	LC50	33.549	Fish	ChV	3.635
			Daphnid	48-hr	LC50	12.531	Daphnid	ChV	2.382
			Green Algae	96-hr	EC50	54.986*	Green Algae	ChV	25.674
			Fish (SW)	96-hr	LC50	13.822	-	-	-
			Earthworm	14-day	LC50	265.356*	-	-	-
			Lemna gibba	7-day	EC50	21.268	-	-	-
		Amides	Fish	96-hr	LC50	60.668*	Fish	ChV	0.139
			Daphnid	48-hr	LC50	53.448*	Daphnid	ChV	4.126
			Green Algae	96-hr	EC50	2.118	Green Algae	ChV	2.440
			Fish (SW)	96-hr	LC50	53.850*	-	-	-
			Mysid (SW)	96-hr	LC50	3.967	-	-	-
		Phenol Amines	Fish	96-hr	LC50	10.619	Fish	ChV	0.432
			Daphnid	48-hr	LC50	1.780	Daphnid	ChV	0.314
			Green Algae	96-hr	EC50	3.652	-	-	-
		Pyrazoles/Pyrroles	Fish	96-hr	LC50	3.778	Fish	ChV	1.152
			Daphnid	48-hr	LC50	9.595	Daphnid	ChV	0.00174
			Green Algae	96-hr	EC50	7.436	Green Algae	ChV	2.204
7	Not readily biodegradable; biodegradable in months	Phenols	Fish	96-hr	LC50	33.549	Fish	ChV	3.635
			Daphnid	48-hr	LC50	12.531	Daphnid	ChV	2.382
			Green Algae	96-hr	EC50	54.986*	Green Algae	ChV	25.674
			Fish (SW)	96-hr	LC50	13.822	-	-	-
			Earthworm	14-day	LC50	265.356*	-	-	-
			Lemna gibba	7-day	EC50	21.268	-	-	-
		Amides	Fish	96-hr	LC50	60.668*	Fish	ChV	0.139
			Daphnid	48-hr	LC50	53.448*	Daphnid	ChV	4.126
			Green Algae	96-hr	EC50	2.118	Green Algae	ChV	2.44
			Fish (SW)	96-hr	LC50	53.850*	-	-	-
			Mysid (SW)	96-hr	LC50	3.967	-	-	-
		Phenol Amines	Fish	96-hr	LC50	10.619	Fish	ChV	0.432
			Daphnid	48-hr	LC50	1.78	Daphnid	ChV	0.314

			Green Algae	96-hr	EC50	3.652	-	-	-
		Pyrazoles/Pyrroles	Fish	96-hr	LC50	3.778	Fish	ChV	1.152
			Daphnid	48-hr	LC50	9.595	Daphnid	ChV	0.00174
			Green Algae	96-hr	EC50	7.436	Green Algae	ChV	2.204
8	Not readily biodegradable; recalcitrant	Amides	Fish	96-hr	LC50	5.700*	Fish	ChV	0.031
			Daphnid	48-hr	LC50	2.561*	Daphnid	ChV	0.519*
			Green Algae	96-hr	EC50	0.273	Green Algae	ChV	0.582*
			Fish (SW)	96-hr	LC50	5.135*	-	-	-
			Mysid (SW)	96-hr	LC50	0.600*	-	-	-
		Pyrazoles/Pyrroles	Fish	96-hr	LC50	0.204	Fish	ChV	0.162
			Daphnid	48-hr	LC50	5.073*	Daphnid	ChV	0.00156
			Green Algae	96-hr	EC50	0.687*	Green Algae	ChV	0.404
9	Not readily biodegradable; biodegradable in months	Amides	Fish	96-hr	LC50	40.617*	Fish	ChV	0.103
			Daphnid	48-hr	LC50	33.054*	Daphnid	ChV	2.859
			Green Algae	96-hr	EC50	1.472	Green Algae	ChV	1.823
			Fish (SW)	96-hr	LC50	36.116*	-	-	-
			Mysid (SW)	96-hr	LC50	2.809	-	-	-
		Pyrazoles/Pyrroles	Fish	96-hr	LC50	2.369	Fish	ChV	0.809
			Daphnid	48-hr	LC50	7.874	Daphnid	ChV	0.00152
			Green Algae	96-hr	EC50	4.969	Green Algae	ChV	1.596
10	Not readily biodegradable; biodegradable in months	Amides	Fish	96-hr	LC50	15.264*	Fish	ChV	0.057
			Daphnid	48-hr	LC50	9.230*	Daphnid	ChV	1.222
			Green Algae	96-hr	EC50	0.636	Green Algae	ChV	1.033
			Fish (SW)	96-hr	LC50	13.661*	-	-	-
			Mysid (SW)	96-hr	LC50	1.302	-	-	-
		Pyrazoles/Pyrroles	Fish	96-hr	LC50	0.697	Fish	ChV	0.363
			Daphnid	48-hr	LC50	6.340*	Daphnid	ChV	0.00154
			Green Algae	96-hr	EC50	1.853	Green Algae	ChV	0.805
11		Amides	Fish	96-hr	LC50	12.413*	Fish	ChV	0.051
			Daphnid	48-hr	LC50	6.994*	Daphnid	ChV	1.024

	Not readily biodegradable; recalcitrant		Green Algae	96-hr	EC50	0.534	Green Algae	ChV	0.927
			Fish (SW)	96-hr	LC50	11.127*	-	-	-
			Mysid (SW)	96-hr	LC50	1.113	-	-	-
		Pyrazoles/Pyrroles	Fish	96-hr	LC50	0.535	Fish	ChV	0.308
			Daphnid	48-hr	LC50	6.182*	Daphnid	ChV	0.00159
			Green Algae	96-hr	EC50	1.505	Green Algae	ChV	0.702
12	Not readily biodegradable; biodegradable in months	Amides	Fish	96-hr	LC50	87.985*	Fish	ChV	1.531
			Daphnid	48-hr	LC50	89.783*	Daphnid	ChV	0.00154
			Green Algae	96-hr	EC50	2.867	Green Algae	ChV	2.763
			Fish (SW)	96-hr	LC50	77.846*	-	-	-
			Mysid (SW)	96-hr	LC50	5.186	-	-	-
		Pyrazoles/Pyrroles	Fish	96-hr	LC50	6.184	Fish	ChV	0.166
			Daphnid	48-hr	LC50	9.544	Daphnid	ChV	5.616
			Green Algae	96-hr	EC50	10.824	Green Algae	ChV	2.887
13	Not readily biodegradable; biodegradable in months	Amides	Fish	96-hr	LC50	87.985*	Fish	ChV	1.531
			Daphnid	48-hr	LC50	89.783*	Daphnid	ChV	0.00154
			Green Algae	96-hr	EC50	2.867	Green Algae	ChV	2.763
			Fish (SW)	96-hr	LC50	77.846*	-	-	-
			Mysid (SW)	96-hr	LC50	5.186	-	-	-
		Pyrazoles/Pyrroles	Fish	96-hr	LC50	6.184	Fish	ChV	0.166
			Daphnid	48-hr	LC50	9.544	Daphnid	ChV	5.616
			Green Algae	96-hr	EC50	10.824	Green Algae	ChV	2.887
14	Not readily biodegradable; biodegradable in months	Amides	Fish	96-hr	LC50	364.327	Fish	ChV	0.401
			Daphnid	48-hr	LC50	565.074*	Daphnid	ChV	19.399
			Green Algae	96-hr	EC50	9.759	Green Algae	ChV	6.698
			Fish (SW)	96-hr	LC50	319.388	-	-	-
			Mysid (SW)	96-hr	LC50	15.975	-	-	-
		Pyrazoles/Pyrroles	Fish	96-hr	LC50	36.16	Fish	ChV	4.941
			Daphnid	48-hr	LC50	13.496	Daphnid	ChV	0.00157
			Green Algae	96-hr	EC50	45.291	Green Algae	ChV	7.553

15	Not readily biodegradable; biodegradable in months	Amides	Fish	96-hr	LC50	11.230*	Fish	ChV	0.048
			Daphnid	48-hr	LC50	6.064*	Daphnid	ChV	0.944*
			Green Algae	96-hr	EC50	0.493	Green Algae	ChV	0.889
			Fish (SW)	96-hr	LC50	10.076*	-	-	-
			Mysid (SW)	96-hr	LC50	1.038*	-	-	-
		Pyrazoles/Pyrroles	Fish	96-hr	LC50	0.467	Fish	ChV	0.286
			Daphnid	48-hr	LC50	6.236	Daphnid	ChV	0.00166
			Green Algae	96-hr	EC50	1.36	Green Algae	ChV	0.663
16	Not readily biodegradable; biodegradable in months	Amides	Fish	96-hr	LC50	33.159*	Fish	ChV	0.092
			Daphnid	48-hr	LC50	25.144*	Daphnid	ChV	2.407
			Green Algae	96-hr	EC50	1.242	Green Algae	ChV	1.641
			Fish (SW)	96-hr	LC50	29.530*	-	-	-
			Mysid (SW)	96-hr	LC50	2.411	-	-	-
		Pyrazoles/Pyrroles	Fish	96-hr	LC50	1.825	Fish	ChV	0.689
			Daphnid	48-hr	LC50	7.707	Daphnid	ChV	0.00157
			Green Algae	96-hr	EC50	4.049	Green Algae	ChV	1.398
17	Not readily biodegradable; biodegradable in months	Amides	Fish	96-hr	LC50	7.812*	Fish	ChV	0.040
			Daphnid	48-hr	LC50	3.713*	Daphnid	ChV	0.694*
			Green Algae	96-hr	EC50	0.364*	Green Algae	ChV	0.738*
			Fish (SW)	96-hr	LC50	7.029*	-	-	-
			Mysid (SW)	96-hr	LC50	0.790*	-	-	-
		Pyrazoles/Pyrroles	Fish	96-hr	LC50	0.293*	Fish	ChV	0.214
			Daphnid	48-hr	LC50	6.020*	Daphnid	ChV	0.00177
			Green Algae	96-hr	EC50	0.943*	Green Algae	ChV	0.523*

(LC_{50} - medium lethal concentration (50 %); EC_{50} - medium effective concentration (50 %); ChV - Chronic Value, defined as the geometric mean between no observed effect concentration (NOEC) and lowest observed effect concentration (LOEC), being used as an estimate for chronic toxicity; SW – salt water). **Green:** practically nontoxic (> 100 $mg.L^{-1}$); **yellow:**: slightly toxic (>10 to ≤ 100 $mg.L^{-1}$); **orange:** moderately toxic (>1 to ≤ 10 $mg.L^{-1}$); **red:** very highly toxic (≤ 1 $mg.L^{-1}$).

***ECOSAR note:** Chemical may not be soluble enough to measure this predicted effect. If the effect level exceeds the water solubility by 10-fold, typically no effects at saturation (NES) are reported. Instances with no data are denoted with "-".

Table S4. Summary of the data obtained by ECOSAR for the aprepitant, fiscalin B, and its derivatives.

			Acute		Chronic	
			**Range (mg.mL^{-1})**	**Media (mg.mL^{-1})**	**Range (mg.mL^{-1})**	**Media (mg.mL^{-1})**
**Aprepitant**			0.283 – 2.275	1.138	0.025 - 0.115	0.081
**Compound**	**Most toxic**	**3**	0.004 - 2.489	0.840	0.002 - 0.043	0.019
		**2**	0.046 - 4.016	1.423	0.002 - 0.178	0.080
		**4**				
		**8**	0.204 - 5.073	1.988	0.002 - 0.404	0.189
	**Less toxic**	**5**	17.057 - 48.644	34.212	0.002 - 8.647	4.722
		**14**	13.496 - 45.291	31.649	0.002 - 7.553	4.165
		**12**				
		**13**	6.184 - 10.824	8.851	0.166 - 5.616	2.890
		**Fiscalin B**				
**Organism**		**Fish**	0.004 - 36.936	6.705	0.012 - 5.518	0.853
		**Daphnid**	1.780 - 17.057	7.030	0.002 - 5.616	0.972
		**Green Algae**	0.028 - 48.644	8.483	0.043 - 8.647	2.035

An analysis of the three most and less toxic compounds was made where the range (minimum – maximum) and mean of the compounds were calculated. The same calculation was also performed for each organism.

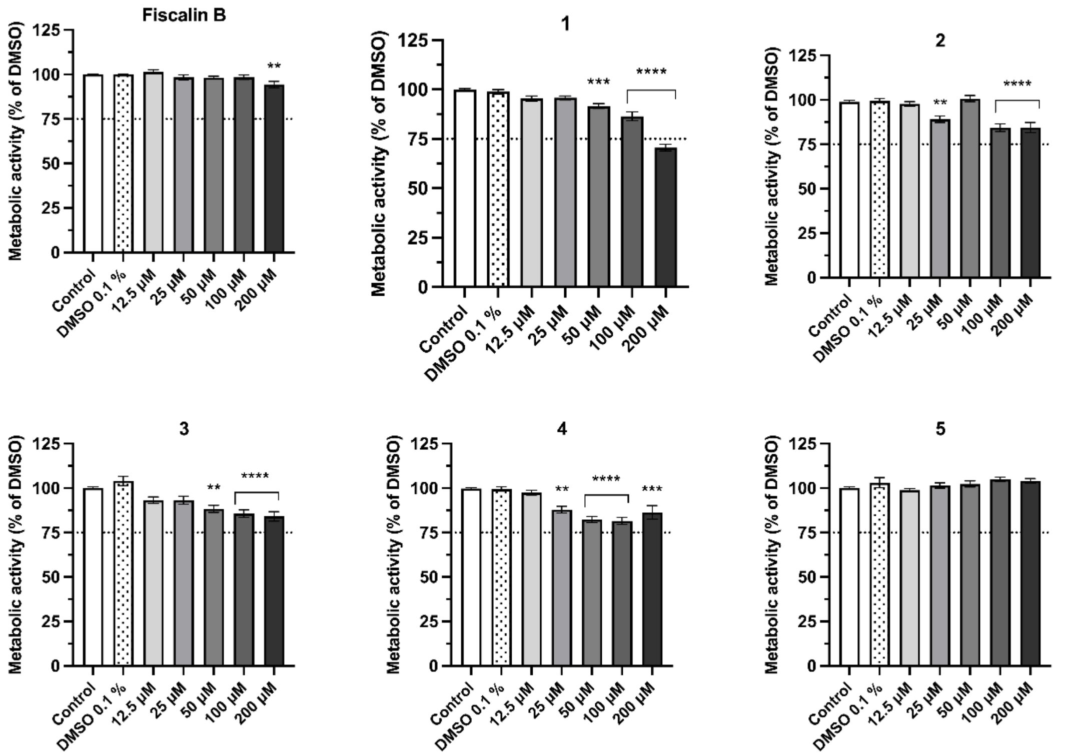

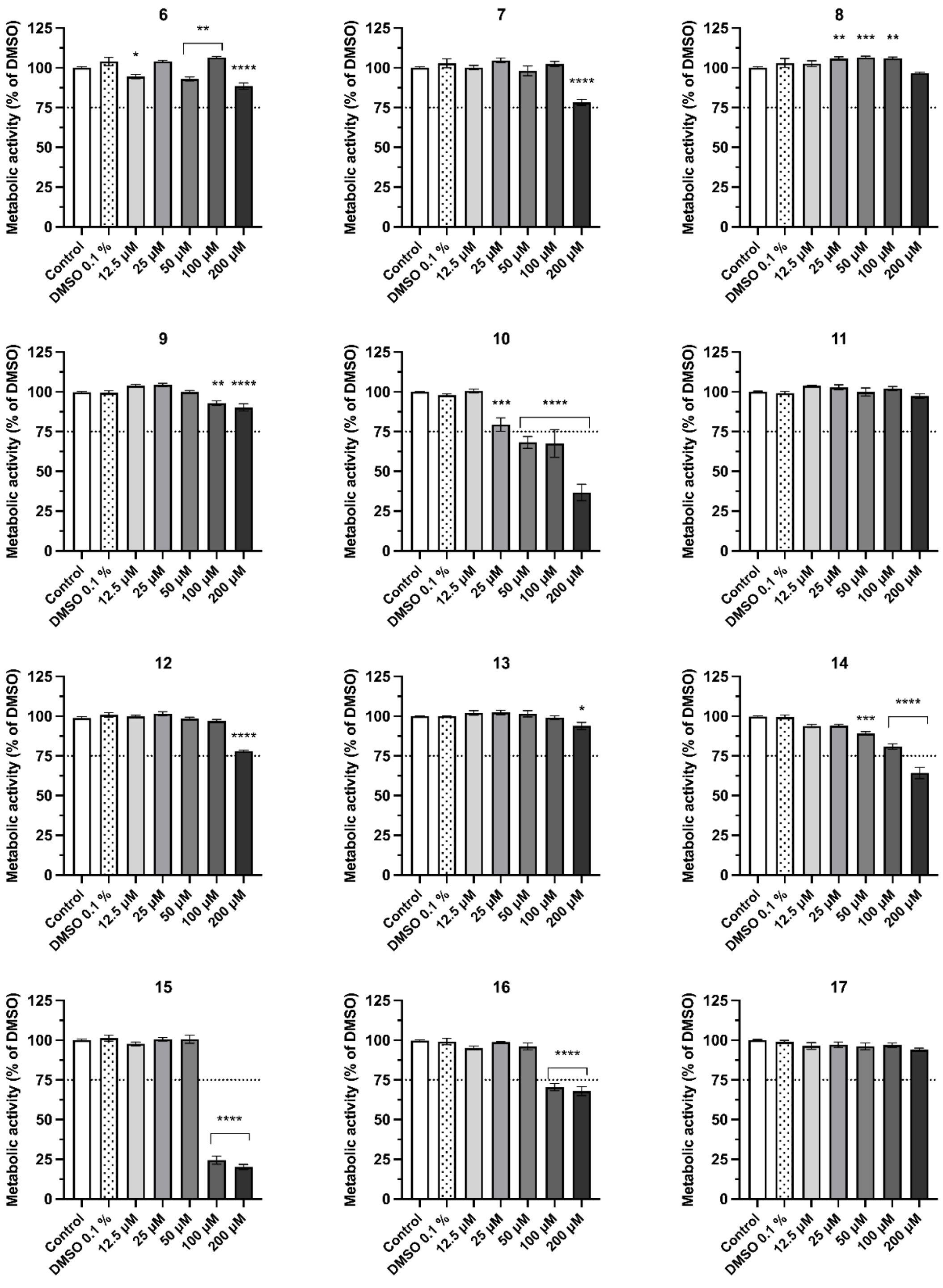

Figure S1. Cell metabolic activity of HaCaT cells exposed to increasing concentrations of fiscalin B and its derivatives (0 – 200 µM) for 24 hours, measured by the Alamar Blue assay. Values are the mean ± SEM of at least four independent experiments performed in duplicate. Statistical comparisons were made using Ordinary One-way ANOVA followed by Dunnett's multiple comparisons test (*$p < 0.05$, **$p < 0.01$, ***$p < 0.001$, ****$p < 0.0001$ *vs* DMSO 0.1 %).

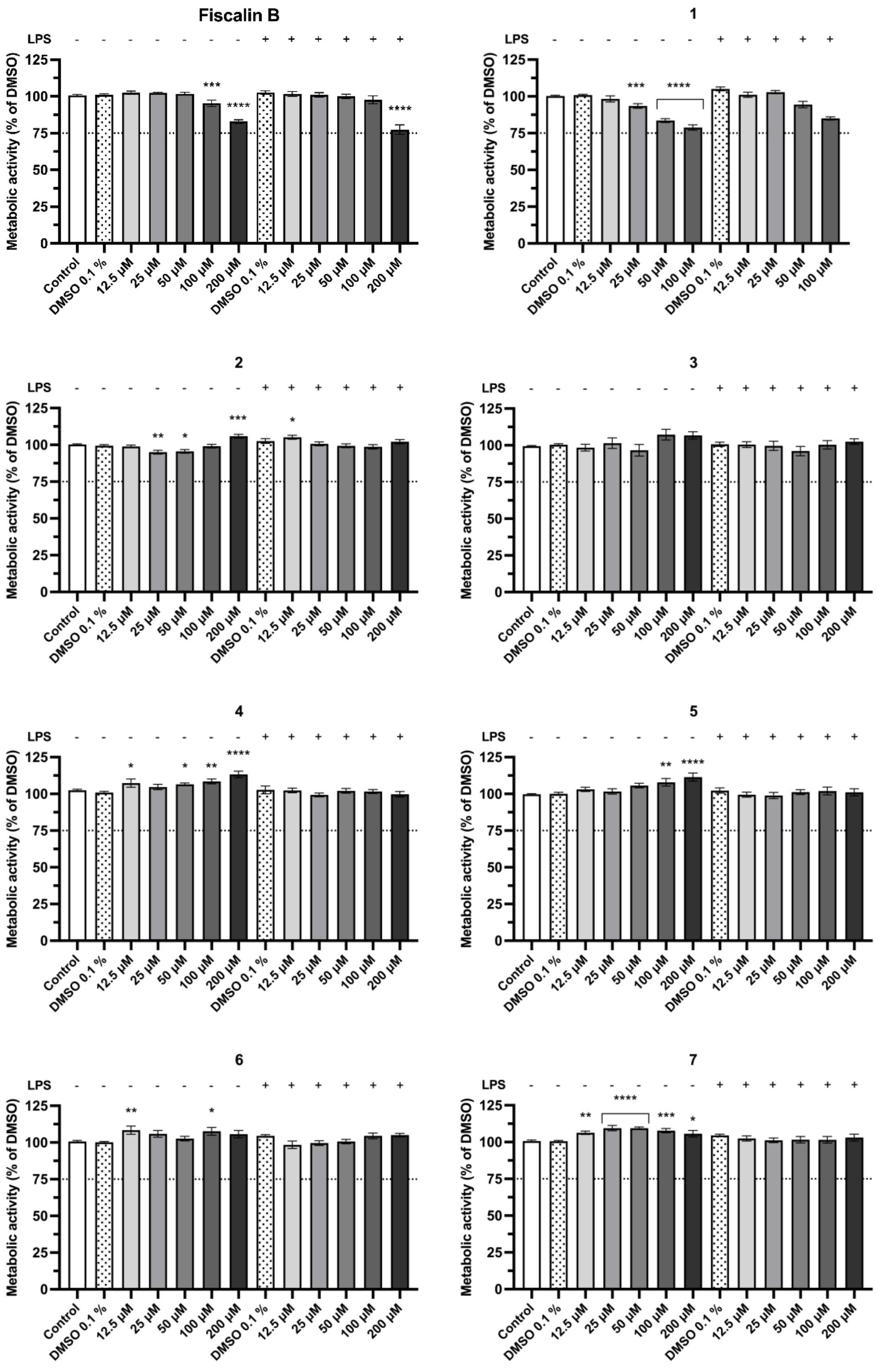
Fiscalin B
1
2
3
4
5
6
7
LPS
Metabolic activity (% of DMSO)
0
25
50
75
100
125
Control
DMSO 0.1 %
12.5 µM
25 µM
50 µM
100 µM
200 µM

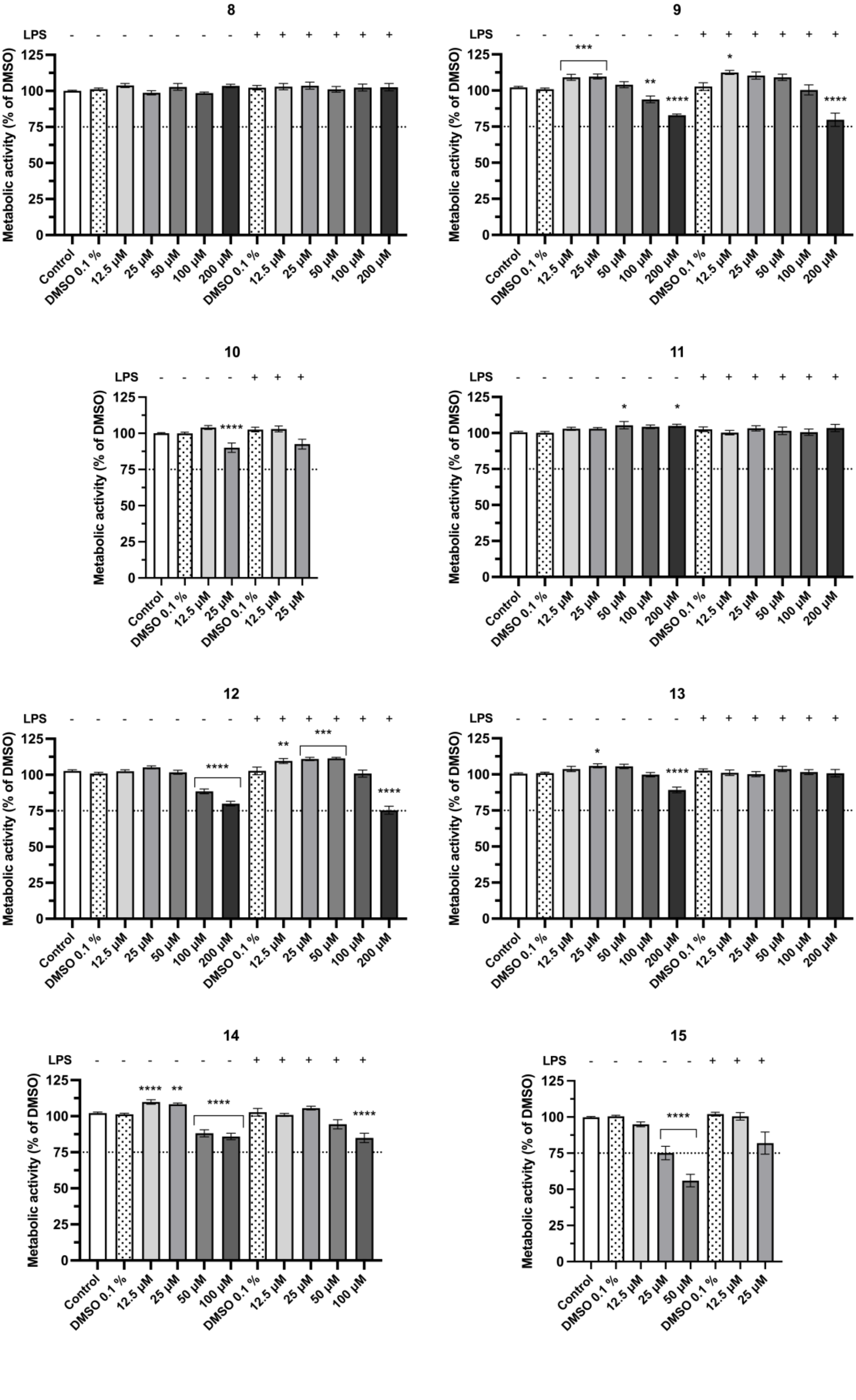
8
9
10
11
12
13
14
15
LPS
Metabolic activity (% of DMSO)
Control
DMSO 0.1 %
12.5 µM
25 µM
50 µM
100 µM
200 µM

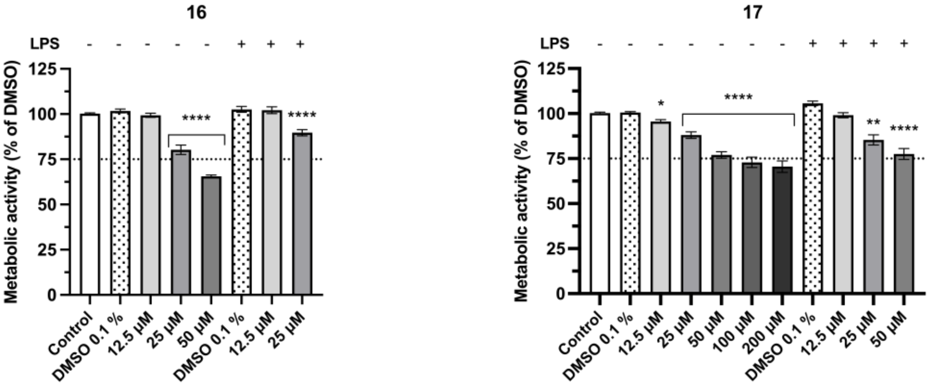

Figure S2. Cell metabolic activity of RAW 264.7 cells exposed to increasing concentrations of fiscalin B and its derivatives (0 – 200 μM) alone or followed by LPS (100 ng.mL^{-1}), for 24 hours, measured by the Alamar Blue assay. Values are the mean ± SEM of at least four independent experiments performed in duplicate. Statistical comparisons were made using Ordinary One-way ANOVA followed by Dunnett's multiple comparisons test (*p < 0.05, **p < 0.01, ***p < 0.001, ****p < 0.0001 *vs* DMSO 0.1 %).

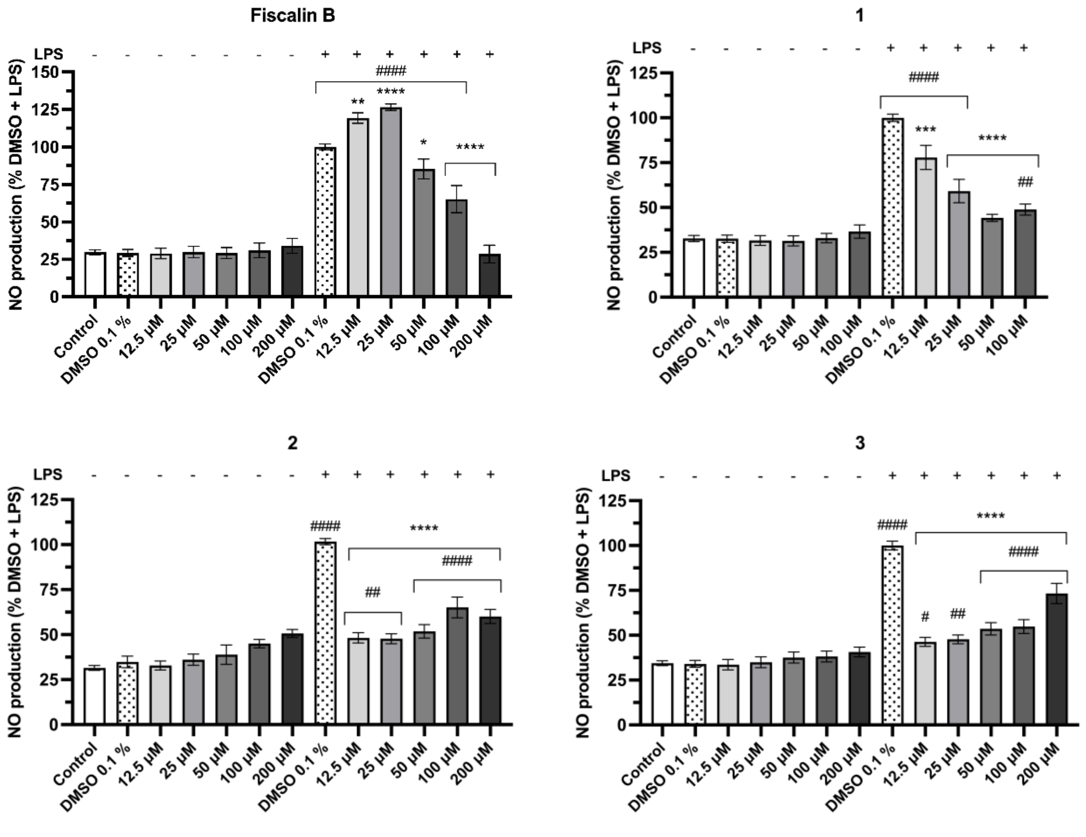

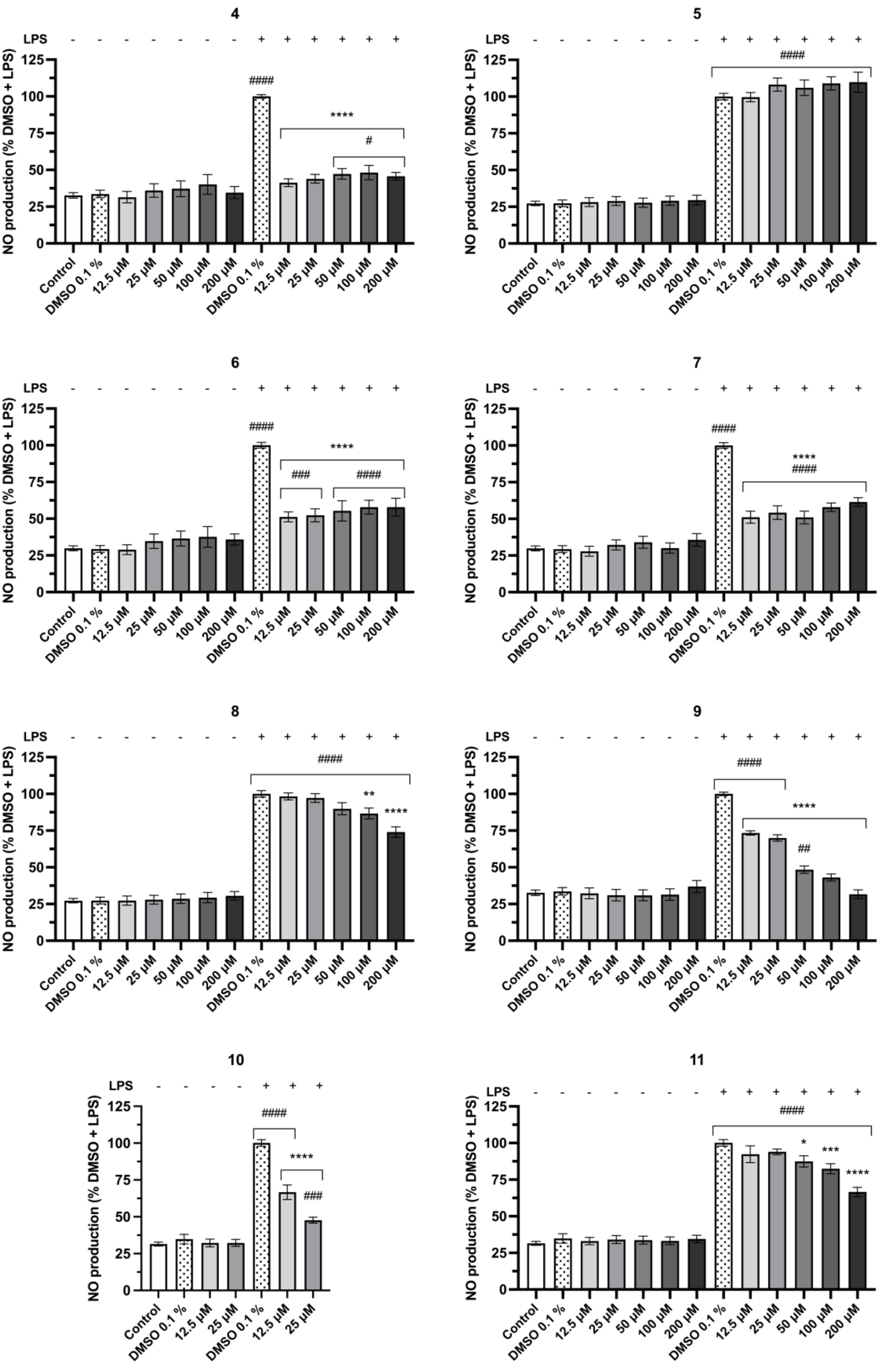
4
5
6
7
8
9
10
11
LPS
NO production (% DMSO + LPS)
Control
DMSO 0.1 %
12.5 µM
25 µM
50 µM
100 µM
200 µM
####
****
###
##
#
***
**
*

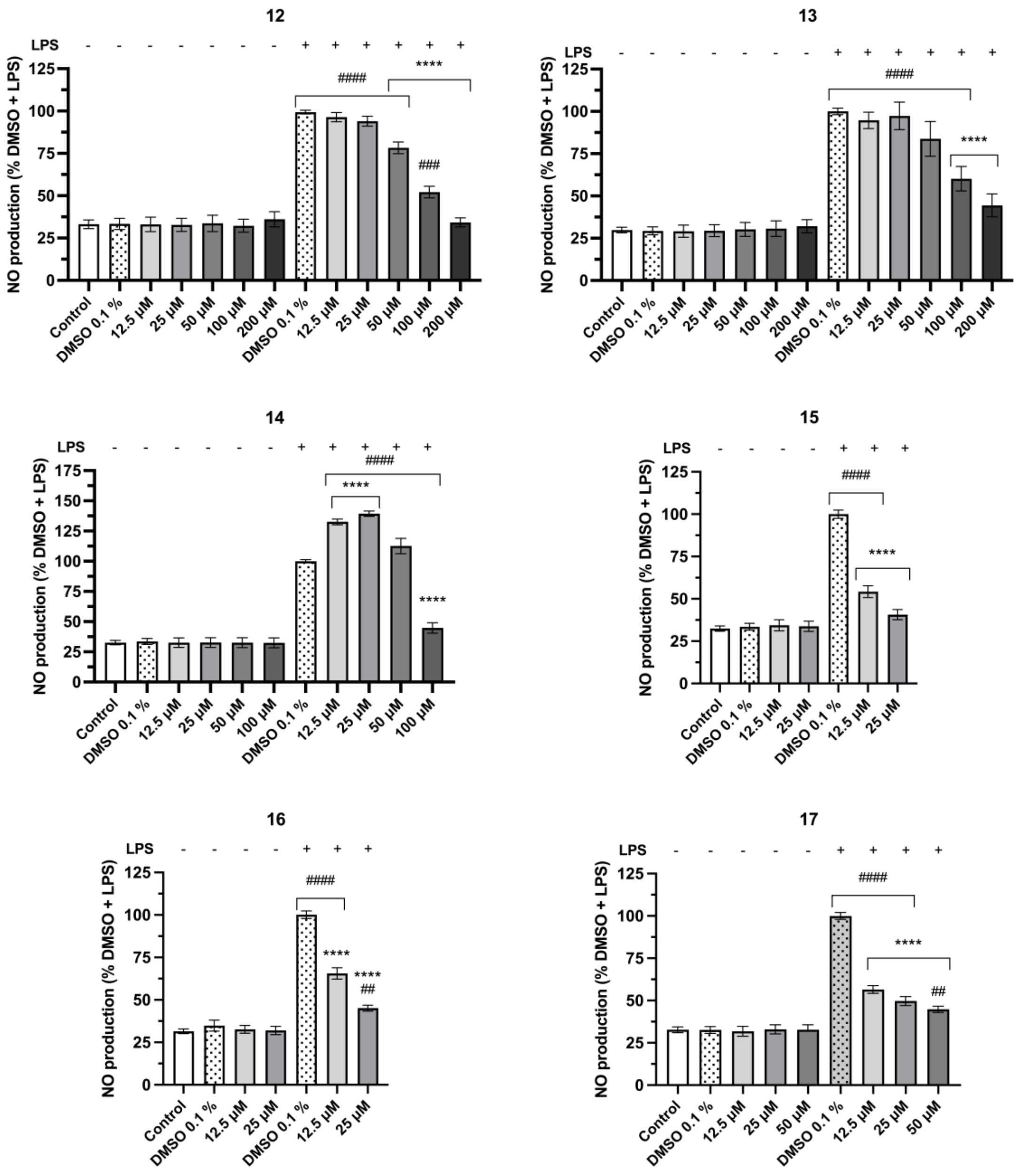

Figure S3. Effect of fiscalin B and derivatives on NO production. Nitrite levels were determined using the Griess assay in RAW 264.7 cells exposed to fiscalin B derivatives (12.5 – 200 µM) in the absence or in the presence of LPS for 24 hours. Values are the mean ± SEM of at least three independent experiments performed in duplicate. Statistical comparisons were made using Ordinary One-way ANOVA followed by Dunnett's multiple comparisons test (****$p < 0.0001$ *vs* DMSO 0.1 % + LPS; $^{\#}p < 0.05$, $^{\#\#}p < 0.01$, $^{\#\#\#}p < 0.001$, $^{\#\#\#\#}p < 0.0001$ *vs* DMSO 0.1 %).
